# The Fusarium crown rot pathogen *Fusarium pseudograminearum* triggers a suite of transcriptional and metabolic changes in bread wheat (*Triticum aestivum* L.)

**DOI:** 10.1093/aob/mcw207

**Published:** 2016-12-07

**Authors:** Jonathan J. Powell, Jason Carere, Timothy L. Fitzgerald, Jiri Stiller, Lorenzo Covarelli, Qian Xu, Frank Gubler, Michelle L. Colgrave, Donald M. Gardiner, John M. Manners, Robert J. Henry, Kemal Kazan

**Affiliations:** 1 Commonwealth Scientific and Industrial Research Organisation (CSIRO) Agriculture, Queensland Bioscience Precinct, St Lucia, 4067 Queensland, Australia; 2 Queensland Alliance for Agriculture and Food Innovation, University of Queensland, 4072, St Lucia, Queensland, Australia; 3 Department of Agricultural, Food and Environmental Sciences, University of Perugia, Borgo XX Giugno 74, 06121 Perugia, Italy; 4 Commonwealth Scientific and Industrial Research Organisation Agriculture, Black Mountain, Australian Capital Territory, 2610, Australia

**Keywords:** Wheat, *Fusarium*, biotic stress, RNA-seq, deoxynivalenol, serotonin, *Tri5*, salicylic acid, jasmonate, secologanin

## Abstract

**Background and Aims** Fusarium crown rot caused by the fungal pathogen *Fusarium pseudograminearum* is a disease of wheat and barley, bearing significant economic cost. Efforts to develop effective resistance to this disease have been hampered by the quantitative nature of resistance and a lack of understanding of the factors associated with resistance and susceptibility. Here, we aimed to dissect transcriptional responses triggered in wheat by *F. pseudograminearum* infection.

**Methods** We used an RNA-seq approach to analyse host responses during a compatible interaction and identified >2700 wheat genes differentially regulated after inoculation with *F. pseudograminearum*. The production of a few key metabolites and plant hormones in the host during the interaction was also analysed.

**Key Results** Analysis of gene ontology enrichment showed that a disproportionate number of genes involved in primary and secondary metabolism, signalling and transport were differentially expressed in infected seedlings. A number of genes encoding pathogen-responsive uridine-diphosphate glycosyltransferases (UGTs) potentially involved in detoxification of the *Fusarium* mycotoxin deoxynivalenol (DON) were differentially expressed. Using a *F. pseudograminearum* DON-non-producing mutant, DON was shown to play an important role in virulence during Fusarium crown rot. An over-representation of genes involved in the phenylalanine, tryptophan and tyrosine biosynthesis pathways was observed. This was confirmed through metabolite analyses that demonstrated tryptamine and serotonin levels are induced after *F. pseudograminearum* inoculation.

**Conclusions** Overall, the observed host response in bread wheat to *F. pseudograminearum* during early infection exhibited enrichment of processes related to pathogen perception, defence signalling, transport and metabolism and deployment of chemical and enzymatic defences. Additional functional analyses of candidate genes should reveal their roles in disease resistance or susceptibility. Better understanding of host responses contributing to resistance and/or susceptibility will aid the development of future disease improvement strategies against this important plant pathogen.

## INTRODUCTION

Fungal pathogens with a necrotrophic phase of their infection cycle, such as *Fusarium* spp., cause substantial losses in grain crops globally, amounting to billions of dollars every year (Savary *et al.*, 2012). Diseases such as Fusarium head blight and root rot, predominantly caused by *F. graminearum* and *F. culmorum*, respectively, pose significant disease threats to wheat and other economically important cereal crops. Fusarium crown rot of wheat, primarily caused by the fungal pathogen *F. pseudograminearum* (Akinsanmi *et al.*, 2006), has high economic significance in Australia, costing the industry in excess of AU$79 million per annum (Murray and Brennan, 2009). This disease is also becoming increasingly important globally (Liu and Ogbonnaya, 2015).

The Fusarium crown rot infection cycle consists of three distinct phases: initial infection in which the pathogen proliferates around the site of infection, a lag phase in which little increase in fungal biomass or symptom development is observed, and a necrotrophic phase in which the pathogen rapidly colonizes internal crown tissue leading to development of necrotic lesions and stem browning (Stephens *et al.*, 2008; Beccari *et al.*, 2011). The pronounced lag phase without disease symptom development has suggested that both *F. pseudograminearum* and *F. graminearum* behave as hemi-biotrophic pathogens during Fusarium crown rot (Kazan *et al.*, 2012).

The basis of host resistance to Fusarium crown rot is relatively poorly understood. Firstly, there is no known wheat genotype that shows complete resistance to this pathogen. Most wheat cultivars infected by Fusarium crown rot develop similar lesion development at the stem base close to maturity and this is promoted by dry conditions. Some wheat cultivars, however, lose less yield under Fusarium crown rot infection, suggesting that they are tolerant to this disease. The available resistance is only partial and is most likely mediated by the action of a large number of genes (Chakraborty *et al.*, 2006). Several quantitative trait loci (QTL) have been identified; however, the degree of resistance contributed by individual QTL is relatively small (Collard *et al.*, 2005; Ma *et al.*, 2010; Poole *et al.*, 2012), limiting their use in plant breeding programmes. Also, different QTL seem to be associated with partial crown rot resistance at different developmental stages (e.g. seedling vs. adult plant resistance) (Martin *et al.*, 2015).

Pathogenic *Fusarium* species also produce an array of trichothecene-based mycotoxins, including deoxynivalenol (DON), nivalenol and T2 toxin (Desjardins, 2006). These toxins can contaminate grains and pose a significant threat to human and animal health (De Boevre *et al.*, 2012). DON is a major component of virulence for *F. graminearum* causing Fusarium head blight with a major resistance QTL (*fhb1*) associated with the ability to detoxify DON (Lemmens *et al.*, 2005). DON also has the ability to trigger reactive oxygen species (ROS) production and defence gene induction in wheat (Desmond *et al.*, 2008*a*), although whether it is a virulence factor during Fusarium crown rot is currently unknown. Previously, DON-responsive uridine-diphosphate glycosyltransferases (UGTs) have been implicated in DON detoxification in wheat and barley (Lulin *et al.*, 2010; Schweiger *et al.*, 2010; Xin *et al.*, 2015). A UGT encoding gene (*HvUGT13248*) from barley (*Hordeum vulgare*) conferred increased tolerance to Fusarium head blight when expressed in wheat (Li *et al.*, 2015). However, so far no wheat UGTs involved in DON detoxification have been functionally characterized.

Functional genomics provides a complementary approach to QTL identification and gene mapping to identify and characterize genes providing resistance to plant pathogens. Wheat genomic resources have improved rapidly with whole shotgun sequencing completed in 2012 (Brenchley *et al.*, 2012) and the release of the chromosome survey sequence in 2014 (Mayer *et al.*, 2014). The genome of the Fusarium crown rot pathogen *F. pseudograminearum* has been sequenced and annotated, enabling pathogenomics approaches to unravel virulence mechanisms on the pathogen side of the interaction (Gardiner *et al.*, 2012). Previous work characterizing the molecular response to a closely related pathogen *F. graminearum* in wheat have utilized RNA-seq approaches to identify host genes and processes responding during infection (Foroud *et al.*, 2012; Xiao *et al.*, 2013) as well as identifying metabolic pathways responding during infection and demonstrating correlation between transcriptional changes observed and accumulation of associated compounds using metabolite quantification approaches (Erayman *et al.*, 2015; Nussbaumer *et al.*, 2015).

It is becoming evident that not all responses induced in the host during a plant–pathogen interaction are beneficial to the host. In some cases, pathogens deliberately activate or hijack certain host responses associated with disease susceptibility (reviewed by Kazan and Lyons, 2014). In fact, a number of disease susceptibility (S) genes have so far been identified from different plant species (reviewed by van Schie and Takken, 2014). For instance, infection of Arabidopsis by the root-infecting pathogen *Fusarium oxysporum* strongly activates certain aspects of the plant hormone jasmonate (JA) signalling, which in turn provides increased susceptibility to infection by this pathogen (Thatcher *et al.*, 2009). Inactivation of the genes involved in disease susceptibility can potentially improve disease resistance, provided that such genes are not involved in other essential plant processes (van Schie and Takken 2014).

In this study, next-generation sequencing approaches were utilized to assess global gene expression responses to *F. pseudograminearum* in a wheat genotype that is considered moderately susceptible in the field as compared to very susceptible wheat genotypes. Differential regulation of genes and synthesis of metabolites involved in biotic stress responses, primary and secondary metabolism, signalling and transport were observed. Genes putatively involved in early signalling of pathogen response such as transcription factors and receptor-like kinases were identified. Genes encoding UGTs similar to those involved in DON detoxification were also activated by pathogen infection. Furthermore, through production of DON-deficient *F. pseudograminearum* mutants, we found that DON is an important virulence factor during Fusarium crown rot. Induction of genes encoding key defence-related metabolites derived from phenylalanine and tryptophan metabolic pathways was observed, indicating tryptamine and serotonin levels were highly induced during Fusarium crown rot infection. Developing a better understanding of the molecular mechanisms that may condition resistance or susceptibility to this pathogen will guide future efforts to improve Fusarium crown rot resistance in wheat.

## MATERIALS AND METHODS

### Infection assay

A soil-less infection assay was performed using the commercial wheat (*Triticum aestivum* L.) ‘Chara’ to observe global transcriptional change during infection by *F. pseudograminearum* isolate CS3427 (CSIRO *Fusarium* collection). *Fusarium pseudograminearum* spores were produced in Campbell’s V8 broth by inoculating with agar plugs taken from *F. pseudograminearum* plate culture and incubating on an orbital shaker at room temperature (∼22 °C) for 1 week. Spores were harvested by filtering culture through Miracloth (Calbiochem, San Diego, CA, USA) and centrifuging filtrate in 50-mL Falcon tubes using a Sigma 4K15 benchtop centrifuge to pellet spores. Spores were suspended in distilled water to a final concentration of ∼1 × 10^6^ spores mL^−1^ and stored at 20 °C until required. Seedlings (3 d post-germination) were immersed in *F. pseudograminearum* spores (1 × 10^6^ spores mL^−1^) and incubated for 3 min. Four biological replicates for *F. pseudograminearum* and mock inoculated seedlings were produced by pooling tissue from approx. 12 plants to correct for inherent biological variation between plants during infection. In order to observe transcriptomic change at a relatively early point during infection, tissue was harvested 3 d post inoculation (dpi) and coleoptile tissue for each plant was excised and immediately immersed in liquid nitrogen.

Validation of successful infection was performed in two ways: firstly, infection replicates were included in the trial and were observed at 14 dpi for development of symptoms. Secondly, cDNA synthesis was performed on aliquots of RNA and relative expression of marker genes for defence responses was assessed using real-time PCR (RT-PCR) with results provided in Supplementary Data File S1 and primers given in Supplementary Data File S2. For all quantitative (q)RT-PCR experiments, Student’s *t* tests (two-sided and assuming equal variance in samples) were applied to compare differences in mean expression values between mock- and *F. pseudograminearum*-inoculated with a *P* value of < 0·05 set as the lower threshold for statistical significance. Having observed a strong molecular response at this time-point, RNA samples were sent to the Ramaciotti Centre (Randwick, New South Wales, Australia) for library preparation and sequencing.

### RNA-seq analysis

RNA extraction was performed using a Qiagen RNeasy extraction kit as per the manufacturer’s instructions with the option for on-column DNase I (Qiagen) digestion. RNA concentration was initially determined using a Nanodrop 2100 spectrophotometer. Integrity of RNA samples was determined using an Agilent Bioanalyser (performed by Australian Genome Sequencing Facility) with all samples with RIN score >8·5. cDNA synthesis was performed using Invitrogen Superscript III cDNA synthesis kit using Oligo-dT primers to promote transcription of whole mRNA molecules according to the manufacturer’s instructions.

In brief, poly-A tail mRNA selection was adopted prior to fragmentation, adaptor ligation, size selection of fragments and amplification. An Illumina HiSeq platform was used to generate 100-bp paired end (PE) reads from isolated mRNA extracted from *F. pseudograminearum* and mock-inoculated biological replicates yielding approx. 160 million reads (35 Gb) in total (read counts for individual libraries given in Supplementary Data File S3). Gene expression based on normalized read counts was reported by DEseq for 66809 coding sequences. Sequence files were deposited at the National Centre for Biotechnology Information (NCBI) Sequence Read Archive under BioProject ID PRJNA297822.

To exclude sequencing errors where possible, sequence quality was analysed using SolexaQA (Cox *et al.*, 2010) and PE reads were trimmed to ensure PHRED score >30 prior to alignment (minimum read length 70 bp). Reads were aligned to the *T. aestivum* Chromosomal Survey Sequence cDNA collection using Bowtie2 with standard parameters (Langmead and Salzberg, 2012). Analysis of differential expression was performed using DEseq. The Chromosomal Survey Sequence was obtained from http://plants.ensembl.org/index.html on 14 May 2014. PE reads were utilized to help solve ambiguous read alignments. On average, 66 % of total reads were successfully aligned to reference across samples. Alignment of global reads to the *F. pseudograminearum* genome reference (Gardiner *et al.*, 2012) failed to produce a significant degree of alignment, indicating a low abundance of *F. pseudograminearum* mRNA within infection samples. This prevented observation of the mixed host–pathogen transcriptome within this study.

Differentially expressed genes were identified using the DESeq analysis package (Anders and Huber, 2012) using standard parameters with an adjusted *P* value of < 0·05 applied as the threshold for statistical significance. qRT-PCR was adopted to confirm expression of a subset of genes across functional categories of interest to confirm that DE genes within the RNA-seq were DE within an independent experiment. qRT-PCR was performed as described by Desmond *et al.* (2006) with new primers designed using Primer3 software and listed in File S2.

### Gene ontology assignment and enrichment testing

BLAST2GO was used to assign annotations and test for as described by Conesa *et al.* (2005). The global coding sequence collection (∼99000 sequences) was analysed using the BLAST2GO server based at CSIRO Agriculture. The global annotated collection was used as a background reference for enrichment analysis with up-regulated genes (>2-fold) used as the test set. The Fisher’s Exact Test enrichment module was used with standard parameters.

### *TRI5* mutant generation and inoculation assays

The targeting construct for deletion of *F. pseudograminearum TRI5* was constructed by amplifying a 4·7-kb fragment containing the *TRI5* gene (*FPSE_12160*) from genomic DNA of isolate CS3096 using FpTRI5KO-f (5′-tcaagcgattgtgttcttgc-3′) and FpTRI5KO2-r (5′-gaagctttttgcggtcagtc-3′). This product was cloned in pCR8/GW-TOPO according to the manufacturer’s instructions (Life Technologies, Carlsbad, CA, USA). The resulting construct was transformed into *E. coli* DY380 to enable recombination-mediated replacement of 1110 bp of the *TRI5* gene with the dual bacterial/fungal antibiotic resistance cassette amplified using TRI5NeoF (5′-taatcattttaataacagaccagtatttctcctaacgagtacccgcaaaACGGCGTAACCAAAAGTCAC-3′) and TRI5NeoR (5′-taccgagacagcaattacacccgagaggagcgcatcgagaacttgcactaCAAGCTTTAACCTGAGGCTATG-3′) primers from pAN9·1 as described previously (Gardiner *et al.*, 2009). Transformation of isolate CS3096 was carried out as previously described (Gardiner *et al.*, 2012). Transformants were screened for deletion of the *TRI5* locus using a three-primer PCR on DNA extracted using the RED extract n amp kit (Sigma, St Louis, MO, USA). The PCR was designed to detect the presence of the wild-type locus (457-bp product) and vector (680-bp product) making use of a common primer (TRI5KOscreen1, 5′-gggtcggggtttagactagc-3′) and template-specific primers (gpdAR for the vector 5′-gagctcacgagttcgtcaca-3′ and TRI5KOscreen2 5′-ggatacagaggacgccaaga-3′ for the wild-type locus). Absence of the wild-type product and presence of the larger vector-specific product was used as an indication of successful deletion of the *TRI5* gene (Supplementary Data File S4). Virulence assays were performed as previously described using wheat cultivar ‘Kennedy’ (Gardiner *et al.*, 2012).

*LC-MS quantification of metabolites*

Some of the coleoptile tissue from the infection assay which had been flash-frozen was also used for metabolite analysis. The samples were ground to a fine powder using a Retsch ball mill and metabolites were extracted with 100 % methanol overnight at room temperature before adding an equal volume of milliQ water and vortexing. Samples were then centrifuged using an Eppendorf 5424 benchtop microcentrifuge at 10000*g* for 10 min. Aliquots (500 µL) of the supernatant were transferred to clean tubes and centrifuged again to pellet protein and cellular debris with the supernatant used for analysis. Extracted plant samples were subjected to liquid chromatography multiple reaction monitoring mass spectrometry (LC-MRM-MS). Five microlitres of each sample was injected into a Shimadzu Nexera UHPLC device and passed through a Kinetex C18 1·7-µm column (Phenomenex 2·1 mm × 100 mm) at 0·4 mL min^−1^ over 15 min at 60 °C with the following gradient: 2 % solvent B for 2 min, a linear gradient from 2 to 45 % solvent B over 8 min, a linear gradient from 45 to 80 % over 1 min, followed by 1 min at 80 % solvent B and an equilibration at 2 % B. The mobile phase consisted of solvent A (0·1 % formic acid/99·9 % water) and solvent B (0·1 % formic acid/90 % acetonitrile/9·9 % water). A 6500 QTRAP mass spectrometer (SCIEX, Foster City, CA, USA) operating in positive ionization mode coupled to the UHPLC device was used to detect and quantify metabolites. The MS source parameters were ion spray voltage (IS) 5500 V, curtain gas 35 psi, GS1 40 psi, GS2 50 psi and source temperature 500 °C. Standards for tryptamine, serotonin hydrochloride, benzoxazalin-2-one, 6-methoxy-benzoxazalinone and DON were obtained from Sigma. Supplementary Data File S5 contains MS parameters specific to each metabolite. The data were acquired and processed by Analyst 1·6.2 software. Each metabolite was detected by measuring four precursor-to-product ion (MRM) transitions and quantified by one transition (File S5). Peaks were integrated using MultiQuant 3·0 (SCIEX), the detection limit was set at a signal to noise (S/N) ratio of > 3 and peaks with S/N > 7 were quantified. Standards were calculated from the average of two technical replicates and experimental samples were an average of two technical replicates of four biological replicates. Data were graphed and analysed in Microsoft Excel.

### Extraction of JA and SA

The extraction of JA and salicylic acid (SA) for analysis by liquid chromatography with tandem mass spectrometry (LC-MS/MS) was modified from the protocol of Dave *et al.* (2011). The leaves were frozen in liquid nitrogen and ground using a mortar and pestle. Ground leaf powder (∼100 mg) was weighed into a 2-mL Eppendorf tube (Eppendorf AG, Hamburg, Germany) and 20 mL of the internal standards (1 mg mL^−1^ dihydrojasmonic acid and 2-hydroxybenzoic acid) followed by 950 mL of extraction solvent (70:30, acetone/50 mm citric acid) was added. Tubes were placed on a shaker at 4 °C in the dark for 5 h and then the tubes were left uncapped in a fume hood to allow the acetone layer to evaporate overnight. The JA and SA in the remaining aqueous phase were extracted by partitioning three times with 500 mL of diethyl ether. The ether phase was collected in a 2-mL glass vial (Kinesis Australia Pty, Redland Bay, Qld, Australia) and evaporated using a speedvac until dry. Dried samples were resuspended in 60 % methanol (50 mL) and filtered through a 0·45-mm GHP membrane in a Nanosep MF Centrifuge tube (Pall Co., Port Washington, WI, USA) before LC-MS/MS analysis. In addition, the accuracy of the assay was determined by spiking a known amount of the pure JA, SA and their respective internal standards to the sample (*n* = 8), assaying the mixture as described above, and then comparing the results with the expected results.

### Quantification of JA and SA by LC-MS/MS

JA and SA were quantified using the method reported by Miyazaki *et al.* (2014). In brief, plant hormones were analysed using an optimized Agilent 6530 Accurate-Mass Q-TOF LC/MS device (Agilent Technologies, Santa Clara, CA, USA). Samples were subjected to optimized electrospray ionization in the Jetstream interface in negative polarity under the following conditions: gas temperature 250 °C, drying gas 9 L min^−1^, nebulizer 25 psig (172·4 kpa), sheath gas temperature 250 °C and flow rate 11 L min^−1^, capillary voltage 2500 V, fragmentor 145 V and nozzle voltage 500 V. Samples (7 mL) were injected onto an Agilent ZORBAX Eclipse XDB-C18 column (2·1 × 50 mm; 1·8 mm) held for 45 min at 0·5 °C and analytes eluted with a linear gradient from 10 to 50 % mobile phase B in 8 min, then to 70 % in 4 min (hold for 8 min) at a flow rate of 200 mL min^−1^. Mobile phase A consisted of water containing 0·1 % formic acid and mobile phase B consisted of methanol containing 0·1 % formic acid. The Q-TOF was operated in targeted MS/MS mode using collision-induced dissociation (N_2_ collision gas supplied at 18 psi (124·1 kPa), *m*/*z* 1·3 isolation window) where the MS extended dynamic range was set from *m*/*z* 100 to 1000 at 3 spectra s^−1^ and MS/MS from *m*/*z* 50 to 1000 at 3 spectra s^−1^. Standards for JA, SA and their respective internal standards, dihydrojasmonate and d4-salicylic acid, were used in the optimized LC-MS/MS to determine their retention times and acceptable collision energies to produce product ions relating to the precursor ions. The LC-MS/MS assays for SA and JA were validated over a range of 0·05–5 ng mL^−1^ and calibration curves were created for JA and SA with the internal standards fixed at a known concentration of 0·4 ng mL^−1^ for subsequent quantification of JA and SA, and to determine the limit of detection and limit of quantification for each hormone. Data were acquired for three subsamples for each sample leaf and analysed using Agilent Technologies Masshunter software (ver. B.4.0).

## RESULTS AND DISCUSSION

### Global analysis of the gene expression response of wheat to infection by F. pseudograminearum

Previous studies analysing gene expression in wheat in response to *F. pseudograminearum* infection revealed that similar defence responses were induced in both partially resistant and susceptible wheat genotypes. However, *PR* gene induction was observed earlier and reached higher levels in the partially resistant cultivar than in the susceptible cultivar (Desmond *et al.*, 2008*a*, *b*). In this study, we conducted RNA-seq experiments to globally analyse gene expression patterns in ‘Chara’, a wheat genotype that shows moderate susceptibility to Fusarium crown rot in the field (Wallwork and Zwer, 2009) although at the seedling stage where responses to infection were analysed in this report, no obvious difference between ‘Chara’ and other wheat genotypes could be observed. Nevertheless, the analysis of a partially susceptible cultivar may facilitate the identification of host processes that are associated either with resistance or with susceptibility. Another reason for selecting this cultivar in this study is due to the availability of substantial genetic resources already developed within this background, such as mutagenized populations that facilitate subsequent gene function analysis (Fitzgerald *et al.*, 2010, 2015).

To assess the transcriptional response during pathogen infection, an established laboratory infection assay with four biological replicates was performed to infect wheat seedlings with *F. pseudograminearum* (Yang *et al.*, 2010). In this assay, ‘Chara’ shows a similar lesion development phenotype to most other bread wheat genotypes so far examined. Leaf sheath enclosed tissue was sampled at 3 dpi. An Illumina HiSeq platform was then used to generate 100-bp PE reads from isolated mRNA extracted from *F. pseudograminearum*- and mock-inoculated plants yielding approx. 175 million reads (35 Gb) in total. On average, across samples 66 % of total reads were successfully aligned to the coding sequence collection from the wheat chromosomal survey sequences (CSS). This coding sequence collection facilitates analysis of gene expression in a homoeologue-specific manner. We used Bowtie2 to align reads to the reference (Langmead and Salzberg, 2012) and DESeq to assess differential expression based on read counts (Anders and Huber, 2012). Gene expression based on normalized read counts was reported by DESeq for 66809 coding sequences. In total, 2755 genes were found to be differentially expressed (adjusted *P*-value 0·05) between mock-inoculated and infected conditions with 1806 up-regulated (910 genes induced >2-fold) and 887 down-regulated (37 down-regulated >2-fold) genes (Supplementary Data File S6). Only genes differentially expressed >2-fold are reported in subsequent sections.

As stated above, previous work has analysed the molecular responses to Fusarium crown rot using a microarray at a different time point, in different wheat genotypes and also using a different inoculation procedure, identifying 217 genes induced >1·5-fold at 1 dpi (Desmond *et al.*, 2008*b*). To determine the overlap between *Fusarium* responsive genes identified in this previous study and the current analysis, coding sequences within the CSS reference corresponding to DE genes were identified using BLAST approaches. Sequences for these accessions were retrieved from NCBI and used as BLAST queries against the CSS coding sequence collection to identify corresponding sequences within the reference. Matching sequences in the CSS reference were identified for 162 DE genes identified in the previous study (e-value < 1×E-10). Of these 162 genes, 119 were induced during infection in the current study (data not shown), suggesting that different wheat genotypes respond to Fusarium crown rot similarly. Nevertheless, by observing transcriptional change at a later point during the infection process and using RNA-seq to overcome potential detection limits imposed by finite microarray probe sets, this study identified a substantially increased set of *Fusarium* responsive genes in a wheat cultivar with significant genetic and genomic resources (Fitzgerald *et al.*, 2010, 2015).

**Fig. 1.**
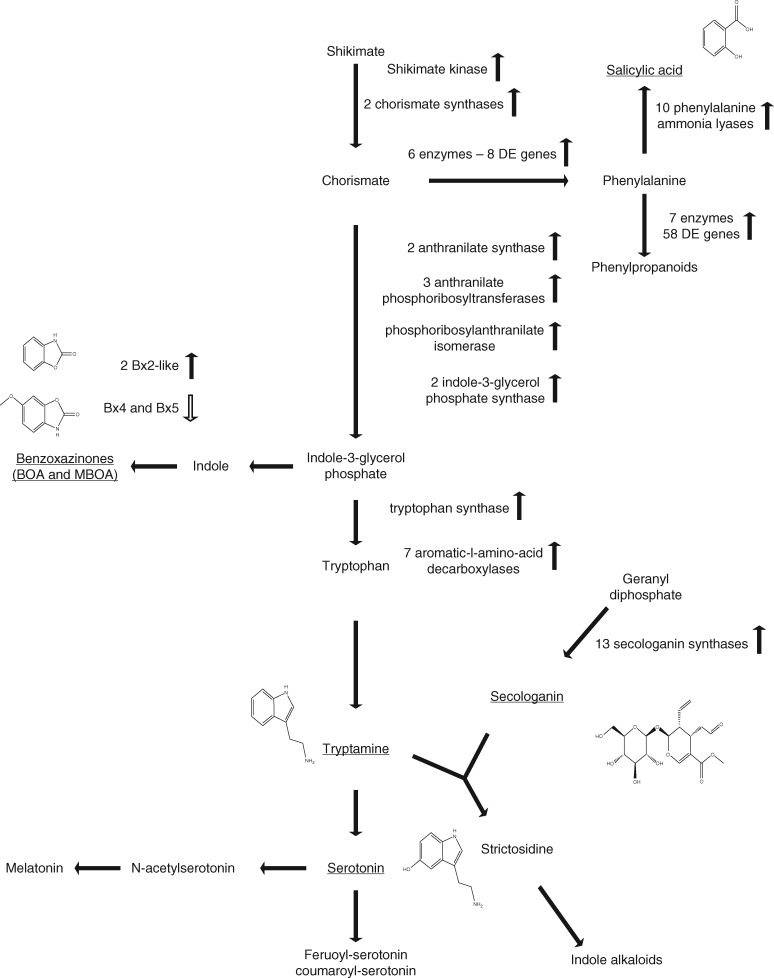
Molecular inference for induction of phenylalanine, tryptamine and tyrosine pathways that lead to the production of defence-associated hormones and metabolites in wheat following *F. pseudograminearum* infection in wheat. The phenylalanine, tyrosine and tryptophan biosynthesis pathway (retrieved from KEGG) denoting *Fusarium* responsive genes encoding enzymes functioning within the pathway. Filled arrows denote up-regulated genes and open arrows denote down-regulated genes. Underlined compound names indicate metabolites which were detected in this study using LC-MS.

The CSS CDS reference was annotated using BLAST2GO (Conesa *et al.*, 2005). Annotations were assigned to 44849 genes representing roughly half of the sequences within the reference. Genes induced >2-fold (910) were queried against the global dataset to determine which gene ontology (GO) terms were enriched in our dataset. In total, 252 biological processes, 146 molecular function and 22 cellular component terms were enriched. Enriched GO categories were associated with biotic stress responses, metabolism, biosynthesis, transport and binding (Supplementary Data File S7). Metabolism- and biosynthesis-associated terms included phytoalexin, flavanol, cinnamic acid and phytosteroid biosynthesis. Specific metabolic terms associated with phytohormone production and signalling were enriched for JA, SA, ethylene and gibberellin (GA) biosynthesis (S7). In addition, using the KEGG pathway mapping feature in BLAST2GO, we identified many enzymes mapping to pentose phosphate and phenylalanine, tryptophan and tyrosine pathways (Fig. 1). Taken as a whole, the set of enriched GO terms are indicative of a host plant in early stages of response to infection: perceiving the pathogen, initiating systemic signalling via phytohormone induction, and responding by deploying chemical and enzymatic defences. To validate RNA-seq results, we performed an infection time-course (1, 3 and 7 dpi) and utilized qRT-PCR to quantify transcript abundance of several selected genes found to be differentially expressed during infection (File S1; see also below).

### Host signalling and response pathways activated by *F. pseudograminearum* infection

#### PR genes.

Thirty-two genes encoding pathogenesis-related (PR) proteins, including six PR1, ten β-1,3-glucanase, four PR5, three PR10 and nine chitinases, were induced during infection (Supplementary Data Table S1). Expression of two PR-encoding genes (PR2 and PR10) and a chitinase induced in the RNA-seq data was also monitored in an independent infection experiment at three different time points using qRT-PCR with all three genes showing induction at 3 and 7 dpi (File S1) consistent with the observations using RNA-seq.

#### Genes for ROS production.

Oxalate oxidase enzymes (also known as germin-like proteins), which catalyse the reaction of oxalate with oxygen and hydrogen ions producing carbon dioxide and hydrogen peroxide radicals (H_2_O_2_), play an important role in mediating ROS responses during pathogen attack (Mittler *et al.*, 2004; Torres *et al.*, 2006). Thirty genes encoding oxalate oxidases and germin-like proteins (Supplementary Data Table S2) were up-regulated in infected samples, suggesting that the activation of ROS responses is part of the plant defence against this pathogen.

#### Genes associated with pathogen sensing and signalling.

Leucine-rich repeat (LRR) receptor-like kinases (LRKs) have been implicated in perceiving pathogens by detecting pathogen associated molecular patterns (PAMPs) in what is termed pattern triggered immunity (PTI) (Jones and Dangl, 2006). Forty LRKs were induced during infection (Supplementary Data Table S3). One hundred non-LRR kinase receptors were also induced by *F. pseudograminearum*, suggesting a large number of signalling cascades are activated during infection. Genes putatively encoding ten disease resistance (R) proteins were induced during infection. This set included five resistance to powdery mildew 1 (RPM1) homologues, two resistant to *Pseudomonas syringae* 2 (RPS2) homologues and three rust resistance homologues (two Lr10 and one Lr21) (Table S3). Resistance proteins, typically possessing nucleotide binding site (NBS) and LRR domains, specifically bind reciprocally matching pathogen-produced effector proteins. The strong induction of these NBS–LRR encoding genes during defence is an indication that they may be playing essential roles in effector recognition. In addition, two disease resistance response protein 206-like and eight MLO1 homologues, a gene conferring susceptibility to barley powdery mildew but resistance to necrotrophic fungal pathogens such as *Ramularia collo-cygni* (McGrann *et al.*, 2014), were up-regulated.

#### Genes encoding transcription factors (TFs).

Thirty TFs including 16 WRKYs, four MYBs, five NACs and three bHLHs, were differentially expressed during infection (Supplementary Data Table S4), suggesting that transcriptional regulation is an important component of the overall defence response. WRKY TFs play important roles in mediating response to both abiotic and biotic stresses (Singh *et al.*, 2002; Chen *et al.*, 2012). In wheat, several WRKY TFs have been previously implicated in disease responses including TaWRKY68 and TaWRKY78 (Proietti *et al.*, 2010; Ding *et al.*, 2014). Other WRKY TFs have been shown to respond strongly to wheat leaf rust (Kumar *et al.*, 2015). In rice, OsWRKY13 mediates resistance to bacterial blight and rice blast through regulation of JA and SA signalling pathways (Qiu *et al.*, 2007). A highly induced WRKY transcription factor was quantified over the infection time-course showing significant induction at 3 and 7 dpi (File S1). MYB TFs have been implicated in mediating defence responses in several plant species. Four genes with close homology to the MYB4 TF of *Arabidopsis*, which mediates resistance to *Hyaloperonospora parasitica*, were induced during infection with induction ranging between 6- and 15-fold. For one of these *MYB* encoding genes, expression was detected only in *F. pseudograminearum*-infected samples. Expression of one *MYB4* TF was independently confirmed by qRT-PCR showing induction at 3 and 7 dpi (File S1).

#### Genes encoding transporters.

Genes encoding transporter proteins (71), particularly ATP-binding cassette (ABC) transporters (34), were also highly represented within the infected dataset (Supplementary Data Table S5), suggesting that infection leads to the transport of several host metabolites and possibly secondary metabolites (Yazaki, 2006). As ABC transporters have also been implicated in transport of conjugated forms of DON from plant cells, these proteins may also play a role in minimizing the effects of pathogen-derived toxins (Mitterbauer and Adam, 2002; Walter *et al.*, 2015). Indeed, in other pathosystems, ABC transporters play important roles in mediating resistance to plant pathogens. For example, one ABC transporter designated as Lr34 provides durable adult resistance to leaf and stripe rust in wheat (Krattinger *et al.*, 2009). Transfer of the genomic *Lr34* sequence from wheat to barley confers resistance to multiple fungal pathogens including barley leaf rust and powdery mildew and in rice provides partial resistance to rice blast (Risk *et al.*, 2013; Krattinger *et al.*, 2015). Three homoeologous PTR2-like peptide transporters, which belong to the PTR class of transporters, were also highly induced. A related orthologue in *Arabidopsis* has been implicated in resistance to bacterial pathogens with knock-out mutants showing increased susceptibility (Karim *et al.*, 2007).

#### UDP glycosyltransferases.

**Fig. 2.**
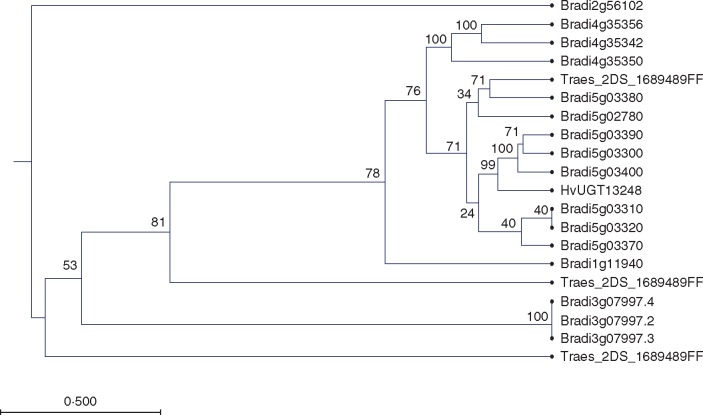
Uridine di-phospate glycosyltransferase (UGT) genes induced during pathogen infection and putatively involved in deoxynivalenol detoxification. Phylogenetic tree of *F. pseudograminearum* responsive wheat UGT genes alongside known deoxynivalenol detoxifying UGT encoding genes from barley (HvUGT13248) and *Brachypodium* (Bradi5g03300). The phylogram shows a redacted version of the phylogram with all *Brachypodium* UDP-glycosyltransferases given in File S4 displaying the clade with deoxynivalenol detoxifying UDP-glycosyltransferases. The phylogeny was produced using the unweighted pair group method (UPGMA) with the Kimura two-parameter model applied. One hundred replicates were performed with bootstrap values displayed at branch nodes. The scale bar represents 0·500 substitutions per nucleotide position

Six UGT encoding genes that were highly up-regulated during infection were identified (Table 1). The homology of the encoded enzymes of these genes was compared to known DON detoxifying enzymes from *Arabidopsis*, barley and *Brachypodium* to determine if these were likely to be functional homologues of the wheat UGTs (Supplementary Data File S8). For this analysis, coding sequences for DE wheat UDP-glycosyltransferases were translated and aligned along with *Brachypodium distachyon* UGT protein sequences analysed in Schweiger *et al.* (2013). Protein sequences for known DON detoxifying enzymes from *Arabidopsis* (AtUGT73C5) (Poppenberger *et al.*, 2003) and barley (HvUGT13248) (Schweiger *et al.*, 2010) were also included in the comparison. This analysis revealed Traes_2DS_1689489FF.1 grouped into the same clade with characterized DON detoxifying enzymes (Fig. 2). In the RNA-seq dataset, Traes_2DS_1689489FF.1 was detected only in *F. pseudograminearum*-infected samples, suggesting that the encoded protein may have a defensive function*.* However, further work is required to determine if these pathogen responsive enzymes possess DON detoxification capability.

**Table 1:** Differentially expressed UGT-encoding genes in wheat in response to infection by *F. pseudograminearum* with IWGSC CSS gene IDs, gene descriptions inferred from BLAST2GO, fold-change values (>2-fold) and adjusted *P* values (*P*-adj < 0·05)

Gene ID	Gene description (BLAST2GO)	DE fold change	*P*-adj
Traes_2DS_1689489FF.1	udp-glycosyltransferase 74f2-like	Inf	0·032
Traes_5AS_067CB4CF9.1	udp-glycosyltransferase 74e1	9·013	0·002
Traes_5BS_D8E13BD7B.1	udp-glycosyltransferase 74f2-like	7·28	1·15E-23
Traes_5DS_19AE064C1.1	udp-glycosyltransferase 74f2-like	7·00	1·15E-08
Traes_2DS_48FB7EC2D.1	udp-glycosyltransferase 74e1	3·80	2·72E-06
Traes_5DL_B1C38ABE0.1	udp-glycosyltransferase 74e1	3·59	0·031
Traes_3B_54AFF431D.1	glycosyltransferase	3·50	0·0052

### DON is a virulence factor for *F. pseudograminearum* towards wheat

A large number of genes induced by *F*. *pseudograminearum* in wheat have been implicated in detoxification processes, including some UGTs that are similar to those implicated in DON detoxification. This suggests that DON detoxification may be an active defence mechanism in wheat against this pathogen. However, the importance of DON as a virulence factor for *F. pseudograminearum* has not been established previously. DON production in *F. graminearum* is encoded and regulated by genes present at four different genetic loci (Kimura *et al.*, 2003). This arrangement at four separate locations, the order of the genes within each locus and the genes immediately flanking the *TRI* genes are conserved in *F. pseudograminearum* with the single exception of the absence of the *TRI7* pseudo-gene in *F. pseudograminearum* (Gardiner *et al.*, 2012).

**Fig. 3.**
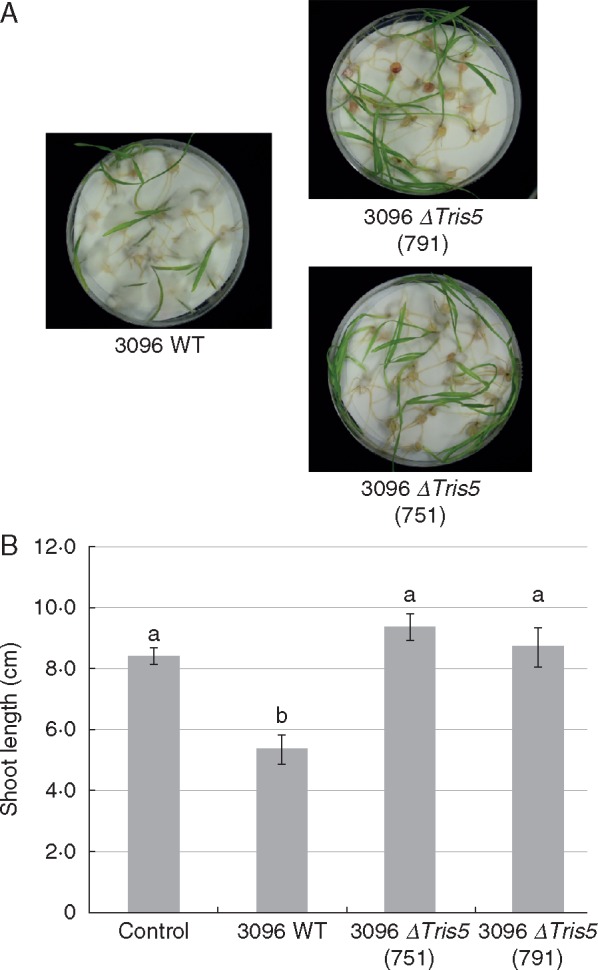
Reduction of virulence in *Tri5 F. pseudograminearum* knockout mutants. (A) Representative infection assays with the parental (CS3096) isolate and two independent mutants. (B) Shoot length is used as a measure of isolate virulence. Plants inoculated with the CS3096 parental strain are significantly shorter than the plants inoculated with the *TRI5* mutants. Student’s *t*-testing was applied to determine statistically significant differences between mean values.

To determine whether DON acts as a virulence factor during wheat–*F. pseudograminearum* interaction, the *TRI5* gene involved in DON biosynthesis was deleted via homologous recombination and replaced with a geneticin resistance cassette in two separate *F. pseudograminearum* strains (CS3096 and CS3427) differing in aggressiveness. Fourteen transformants were generated with two successful targeting events observed for each isolate (File S4). Two Δ*TRI5* strains in each background were used in wheat root rot inoculation assays and the strains in the CS3096 background were found to be less virulent than the parental strain (Fig. 3) in repeated inoculation experiments. Mutants in the CS3427 background were equally pathogenic to their parental strain (data not shown). These results suggest that DON is also a virulence factor for *F. pseudograminearum* when infecting wheat seedlings. The differences observed between isolates of *F. pseudograminearum* suggest many different virulence factors contributing quantitatively to virulence of this pathogen and that in highly aggressive strains the loss of one of these is not as detrimental to the pathogen as it is in a less aggressive isolate. Similar strain-to-strain differences have been observed in *Ustilago maydis* on maize (Djamei *et al.*, 2011). Given the ability of DON to trigger ROS-mediated cell death in wheat (Desmond *et al.*, 2008*b*), it is possible that these dead cells facilitate the initial infection by the pathogen and thus in the absence of DON, pathogen virulence is attenuated, a possibility that requires additional investigation.

### Hormone signalling: JA, SA and GA pathways are induced and cytokinin signalling may be supressed during *F. pseudograminearum* infection of wheat

Both SA and JA function as regulators of defence responses in vascular plants (Bari and Jones, 2009). In *Arabidopsis*, SA and JA interact antagonistically with SA primarily effective against biotrophic pathogens while JA is effective against hemi-biotrophic and necrotrophic pathogens (Spoel *et al.*, 2003; Takahashi *et al.*, 2004). There is a paucity of information on the interaction between SA and JA in monocot cereals and their direct roles in mediating effective resistance against different pathogens (Lyons *et al.*, 2013). Previous work has implicated jasmonate as the primary responsive signal during *F. pseudograminearum* infection (Desmond *et al.*, 2006). The JA/ET ethylene has also been shown to be important for Fusarium head blight resistance in wheat (Gottwald *et al.*, 2012). A typical marker gene for JA biosynthesis, 12-oxophytodienoate synthase (OPR) catalyses a critical step from 12-oxo-*cis*-10,15-phytodienoic acid (OPDA) to 3-oxo-2-(*cis*-2′-pentenyl)-cyclopentant-1-octonoic acid (OPC) in the JA biosynthesis pathway (Kazan and Manners, 2008). Nine *OPR* genes were induced during infection (Table 2), suggesting that JA biosynthesis may be induced at 3 dpi. The JA signalling pathway often works synergistically with ethylene with an integral role played by ethylene responsive transcription factors (e.g. ERFs). One *ERF* encoding gene (Traes_1AL_7BE5906B2.1) was up-regulated 5·3-fold at 3 dpi. Induction of ethylene biosynthesis was also inferred based on up-regulation of 12 aminocyclopropane carboxylate (ACC) oxidases (Supplementary Data Table S6).

**Table 2:** Differentially expressed 12-oxophytodienoate reductase and phenylalanine ammonia lyase encoding genes in wheat in response to infection by *F. pseudograminearum* with IWGSC CSS gene IDs, gene descriptions inferred from BLAST2GO, fold-change values (>2-fold) and adjusted *P* values (*P*-adj < 0·05)

Gene ID	Gene description (BLAST2GO)	DE fold change	*P*-adj
Traes_2DS_632399075.1	phenylalanine ammonia-lyase	3·79	0·00047
Traes_2BS_88CF42F2E.1	phenylalanine ammonia-lyase	3·53	4·36E-13
Traes_2DS_28CA50371.1	phenylalanine ammonia-lyase	3·47	7·61E-08
Traes_1DS_A171C7D59.1	phenylalanine ammonia-lyase	3·46	4·23E-08
Traes_4AL_892C47ED5.1	phenylalanine ammonia-lyase	2·78	3·19E-08
Traes_1AS_6BDC65775.1	phenylalanine ammonia-lyase	2·46	0·0058
Traes_1AS_F9013A945.1	phenylalanine ammonia-lyase	2·36	0·0046
Traes_2DS_3791A8A36.1	phenylalanine ammonia-lyase	2·13	0·00030
Traes_1BS_723922D171.1	phenylalanine ammonia-lyase	2·07	0·000054
Traes_1BS_723922D171.1	phenylalanine ammonia-lyase	2·07	0·000054
Traes_6BL_5B613F9E5.1	12-oxophytodienoate reductase 2	13·22	1·28E-12
Traes_7DS_28E2128F3.1	12-oxophytodienoic acid reductase	10·19	0·015
Traes_6DL_94DCF0B70.1	12-oxophytodienoate reductase 2	7·21	3·14E-05
Traes_1DS_AE7405E32.1	12-oxophytodienoate reductase 1	5·91	0·023
Traes_1DS_D8B54340D.1	12-oxophytodienoate reductase 1	4·07	1·29E-05
Traes_1DS_91D9863D0.2	12-oxophytodienoate reductase 1	3·88	0·0016

Ten phenylalanine ammonia lyase (*PAL*) genes were also up-regulated in the experiment, suggesting SA may be up-regulated by *F. pseudograminearum*; however, the fold-change values were relatively low (Table 2). SA has been shown to be important for resistance to Fusarium head blight (Makandar *et al.*, 2012) possibly due to a direct effect on fungal growth rather than through mediating host defence responses (Qi *et al.*, 2012).

**Fig. 4.**
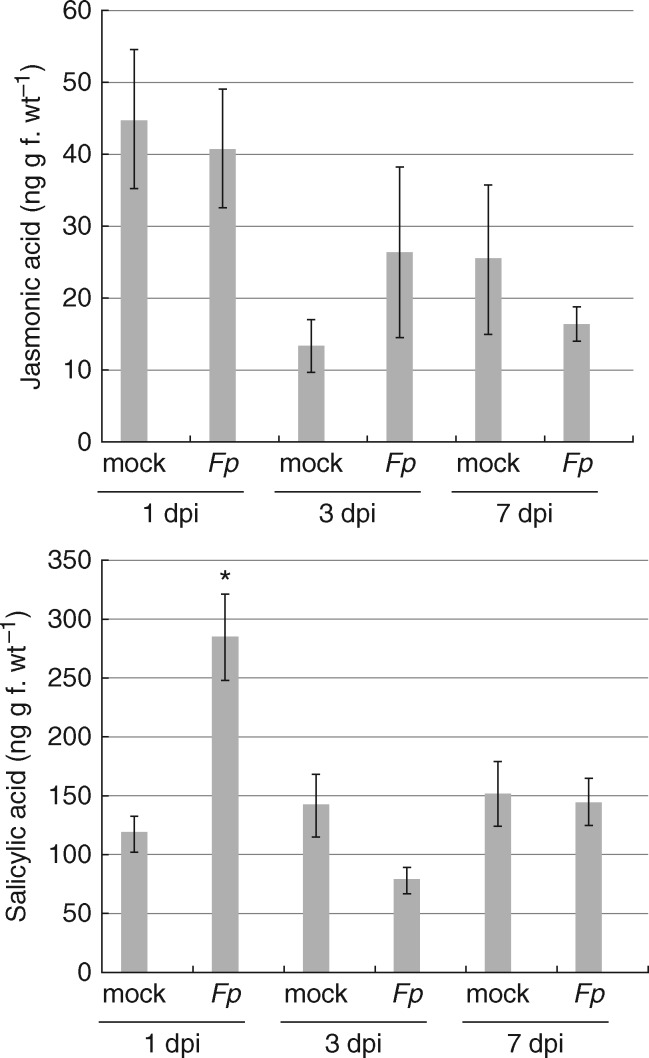
Quantification of salicylic acid and jasmonate in above leaf sheath tissue across an *F. pseudograminerum* infection time-course using LC-MS. Bar graphs denote mean quantification values (ng metabolite per ng tissue) across four biological replicate samples for mock- versus *F. pseudograminearum*-inoculated samples at 3 and 7 dpi. Error bars represent the standard error between biological replicates. Student’s *t*-testing was applied to determine statistically significant differences (*P < 0·05) between mean values.

To determine whether *F. pseudograminearum* infection alters phytohormone levels in wheat, we quantified SA and JA using LC-MS in a time-course experiment. These results showed SA levels were slightly increased at 1 dpi, but not at later time-points, while JA levels did not change at any time-point (Fig. 4). The strong induction of *PR* genes at 3 and 7 dpi in the time-course indicates *F. pseudograminearum*-treated wheat seedlings were responding strongly to infection, but this might be occurring independently of SA or JA induction. Alternatively, hormone levels may have increased rapidly at an earlier time-point to sustain subsequent *PR* gene expression.

Abscisic acid (ABA) plays an integral role in many biological processes and is associated with both resistance and susceptibility to different pathogens (Anderson *et al.*, 2004; Mauch-Mani and Mauch, 2005; Kazan and Lyons, 2014). A small number of genes involved in ABA-related processes were differentially expressed within the dataset, including an ABA-responsive protein that was >20-fold induced (Traes_5BL_0BFAE33B9.1).

Cytokinins also play a role both in regulation of plant growth and in mediating defence responses (Bari and Jones, 2009; Davies, 2010). *Arabidopsis* response regulator (ARR)-like genes, classical markers for cytokinin biosynthesis (D’Agostino *et al.*, 2000), were not differentially expressed during infection in wheat. However, several cytokinin glycosyltransferases were highly induced by *F. pseudograminearum* (Supplementary Data Table S7). Cytokinin glycosyltransferases inactivate cytokinin compounds (e.g. kinetin and zeatin) by adding a glycosyl group (Ostrowski and Jakubowska, 2014) and the activation of these genes suggests infected plants may be responding to high levels of cytokinins. Similarly, GA compounds play a vital role in both plant defence and plant growth and development (Navarro *et al.*, 2008; Ueguchi-Tanaka *et al.*, 2007). Key markers for GA biosynthesis such as GA20 (two genes) and ent-kaurene synthase genes were up-regulated by *F. pseudograminearum*. Three repressor of GA (RGA) homologues were also induced. Whether these pathways are being manipulated by the fungus or are a direct part of the host response is yet to be dissected (Kazan and Lyons, 2014).

### A diverse set of putative phytoalexin biosynthetic pathways are induced under *F. pseudograminearum* infection

Fifty-three cytochrome P450 enzymes were differentially expressed (Supplementary Data Table S8) with the majority annotated within five distinct sub-families, including subfamily 99a2 associated with momilactone (an allelopathic agent) synthesis in rice (Yamamura *et al.*, 2015), subfamily 86b1 associated with suberin biosynthesis (Pinot and Beisson, 2011) and subfamily 71c associated with benzoxazolinone biosynthesis (Nomura *et al.*, 2005). Benzoxazalinone compounds encoded by *Bx* genes are known major phytoalexins produced in wheat (Nomura *et al.*, 2002), maize (Frey *et al.*, 1997) and rye (Bakera *et al.*, 2015). Recent work has shown *F. pseudograminearum* can detoxify benzoxazolinone compounds utilizing a two-step detoxification pathway (Kettle *et al.*, 2015*a*, *b*). This suggests that these compounds may play a role in defence against *F. pseudograminearum*. However, we found that *Bx1*, *Bx2* and *Bx3* were not differentially expressed during infection and *Bx4* and *Bx5* were repressed 2-fold. Two additional genes encoding Bx2-like indole-2-monoxygenase-like genes were highly induced (56- and 12-fold), putatively playing roles catalysing free indole into indolin-2-one.

To assess whether benzoxazalinone levels are altered during infection, we also quantified 6-methoxy-2-benzoxazalinone (MBOA) and 2-benzoxazalinone (BOA) levels using LC-MS. BOA was detected across wheat samples with no patterns of induction or reduction during infection (data not shown). MBOA accumulated to high levels (in both mock-inoculated and infected plants) at both 3 and 7 dpi, suggesting that benzoxazolinone compounds may not form part of induced host defence against this pathogen (Supplementary Data File S9).

### Phenylalanine- and tryptophan-derived metabolic pathways are significantly up-regulated under infection

We identified 12 up-regulated genes encoding four enzymes (EC 2.2.1.2, EC 3.1.3.11, EC 1.1.1.44 and EC 1.1.1.49) involved in production of d-erythrose-4-phosphate, suggesting that the pentose phosphate pathway was altered in infected plants (File S6). d-Erythrose-4-phosphate functions as a primary input to the phenylalanine, tyrosine and tryptophan (PTT) biosynthesis pathway. Among the highly DE genes, one gene functions within the PTT pathway encoding a shikimate kinase metabolizing shikimate to shikimate-3-phosphate and two genes encoding chorismate synthases catalysing the reaction from 5-O-(1-carboxyvinal)-3-phosphoshikimate to chorismate. Chorismate functions as a common precursor to production of phenylalanine, tyrosine and tryptophan and has a primary role in priming and producing host defence responses (Loake and Grant, 2007).

Phenylalanine is an important precursor to key defence metabolites including SA, lignins, flavanoids, anthocyanins, coumarins and tannins (Dixon *et al.*, 2002). Lignin deposition is a known common defence response to pathogen infection (Miedes *et al.*, 2014), particularly against biotrophic fungal pathogens as a barrier to haustorial penetration (Underwood, 2012). Several genes putatively encoding peroxidases associated with production of various lignin compounds were highly up-regulated, providing molecular inference for induction of lignin deposition during early stages of infection. Tryptophan also functions as an important precursor to defence-related compounds, in particular indole-based phytoalexins (Iven *et al.*, 2012). Several genes involved in tryptophan metabolism, including an indole-3-glycerol phosphate synthase potentially performing a step in metabolizing anthranilate to indole-3-glycerol phosphate, were up-regulated during infection by *F. pseudograminearum* (File S6).

**Fig. 5.**
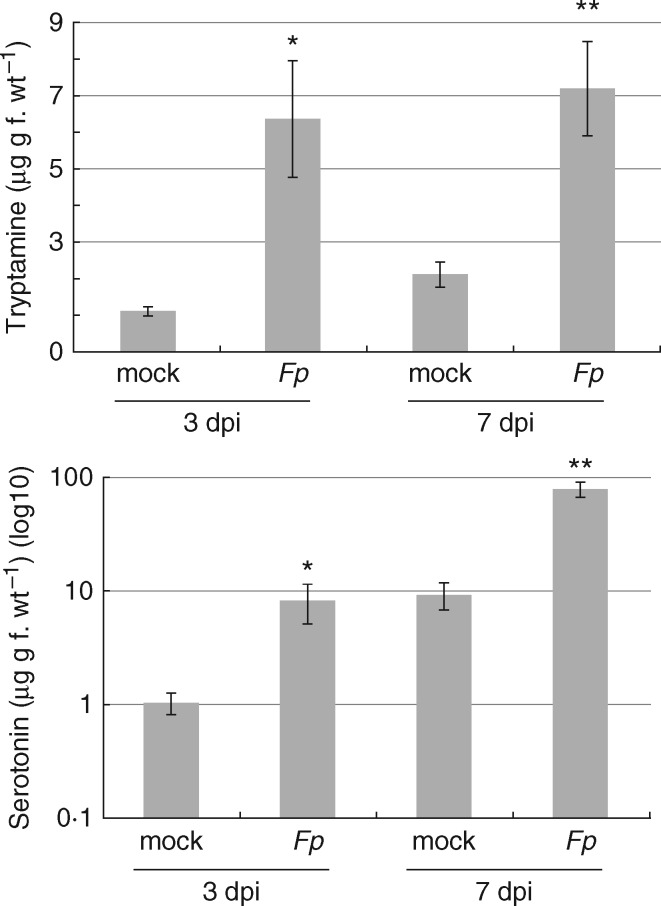
Quantification of tryptamine and serotonin in wheat seedlings across an *F. pseudograminerum* infection time-course using LC-MS. Bar graphs denote mean quantification values (µg metabolite per g tissue) across four biological replicate samples for mock- versus *F. pseudograminearum*-inoculated samples at 3 and 7 dpi. Error bars represent standard error between biological replicates. Student’s *t*-testing was applied to determine statistically significant differences (*P < 0·05) between mean values.

Seven genes putatively encoding aromatic l-amino-acid decarboxylase (AADC) enzymes were highly induced, with expression of five detected only in *F. pseudograminearum-*infected samples (Table 3). Up-regulation of AADC enzymes has been previously correlated with induction of tryptamine and serotonin during Fusarium head blight infection in *Brachypodium* (Pasquet *et al.*, 2014). To determine if the induction of AADC-encoding genes correlates with increases in corresponding metabolite levels, tryptamine and serotonin were quantified using LC-MS. Both tryptamine and serotonin were induced by *F. pseudograminearum* at 3 and 7 dpi (Fig. 5). Tryptamine has been previously shown to inhibit spore germination and appressorium formation of the rice blast-causing fungal pathogen, *Magnaporthe grisea* (Arase *et al.*, 2001). The role of serotonin in plants is poorly understood; however, potential roles suggested include an inhibitor of senescence, a functional analogue for auxin compounds, a phytoalexin or a phytohormone (Akula *et al.*, 2011). Serotonin levels have been shown to be induced during infection by *Parastagonospora nodorum* in wheat (Du Fall and Solomon, 2013) and by *F. graminearum* in *Brachypodium* (Pasquet *et al.*, 2014), suggesting that this compound might play a role in plant defence.

**Table 3:** Differentially expressed aromatic-l-amino acid decarboxylase (AADC) encoding genes in wheat in response to infection by *F. pseudograminearum* with IWGSC CSS gene IDs, gene descriptions inferred from BLAST2GO, fold-change values (>2-fold) and adjusted *P* values (*P*-adj<0·05)

Gene ID	Gene description (BLAST2GO)	DE fold change	*P*-adj
Traes_2BL_424BDA37F.2	aromatic-l-amino-acid decarboxylase	Infinite	0·0061
Traes_2DL_3E4BFB46E.1	aromatic-l-amino-acid decarboxylase	Infinite	0·031
Traes_1BS_1172A6B03.1	aromatic-l-amino-acid decarboxylase	Infinite	0·032
Traes_7DL_E9DA2078F.1	aromatic-l-amino-acid decarboxylase	Infinite	0·034
Traes_2DL_CE7051D49.1	aromatic-l-amino-acid decarboxylase	Infinite	0·041
Traes_2AL_796D6C2DE.1	aromatic-l-amino-acid decarboxylase	41·81	0·0022
Traes_7AL_92598DE70.1	aromatic-l-amino-acid decarboxylase	20·67	0·012

### Putative terpene and secologanin biosynthesis genes are highly Fusarium crown rot responsive

Geranylgeranyl-diphosphate (GGPP) also presents as an important precursor compound for the production of defence-related metabolites (Coman *et al.*, 2014). Four homologues of the class 1 and four homologues of the class 2 terpene synthases involved in rice phytoalexin biosynthesis including terpenes (Schmelz *et al.*, 2014) were induced within infected samples (Supplementary Data Table S9), suggesting production of diterpenoid phytoalexins compounds. In rice, these enzymes catalyse the production of phytoalexin/allelopathic compounds from GGPP (Xu *et al.*, 2004).

GGPP is a precursor for the production of the defence compound secologanin. Biosynthesis of secologanin has primarily been studied in Madagascar periwinkle (*Catharanthus roseus*) due to its role as a precursor for the compounds vinblastine and vincristine, compounds with pharmaceutical significance due to their anti-cancer properties (de Bernonville *et al.*, 2015). To our knowledge, the production of secologanin has not been shown in wheat. However, within the RNA-seq dataset, 13 genes annotated as encoding secologanin synthase (which are cytochrome P450 monooxygenases) were induced (Table 4). To determine if the induction of these putative secologanin biosynthesis genes correlates with the production of this metabolite we used LC-MS to detect and quantify secologanin and found that it was indeed produced in wheat, but not induced at the time-points observed. (Supplementary Data File S10). Several compounds within the monoterpene indole alkaloid class are known to exhibit anti-fungal properties (Joosten and van Veen, 2011; Marei *et al.*, 2012; Tintu *et al.*, 2012). Secologanin production has been previously found to be strongly induced by the exogenous application of methyl jasmonate in *C. roseus*, perhaps suggesting a link between defence-related signalling pathways and monoterpene indole alkaloid production (Zhang *et al.*, 2011). A study using double haploid barley lines differing in Fusarium head blight sensitivity observed metabolite accumulation and found secologanin was constitutively produced in resistant lines (Chamarthi *et al.*, 2014). Secologanin may possess a direct anti-fungal effect or it may be utilized as a precursor to produce other phytoalexins playing a role in mediating defence against fungal pathogens. If secologanin is utilized as a precursor, as it is in the biosynthesis of the alkaloids vinblastine/vincristine in *C. roseus*, this may indicate there are unidentified monoterpene-alkaloids in wheat. Alkaloids are known to play an important role in defence, with compounds such as caffeine, morphine and quinine thought to have evolved in plants to provide resistance against herbivores and pathogens (Ashihara *et al.*, 2008; Ziegler *et al.*, 2009). To date, relatively few alkaloid compounds have been identified within wheat. With a more detailed understanding of how cereal crops and related grass species respond to *Fusarium* pathogens, opportunities to discover and deploy novel mechanisms of resistance will emerge. Identifying the full repertoire of phytoalexins and other anti-fungal metabolites produced by wheat and the degree of sensitivity exhibited by *Fusarium* pathogens could be one route to deriving novel disease resistance strategies.

**Table 4:** Differentially expressed secologanin synthase encoding genes in wheat in response to infection by *F. pseudograminearum* with IWGSC CSS gene IDs, gene descriptions inferred from BLAST2GO, fold-change values (>2-fold) and adjusted *P* values (*P*-adj < 0·05)

Gene ID	Gene description (BLAST2GO)	DE fold change	*P*-adj
Traes_6BL_F4597CA77.1	secologanin synthase	Infinite	0·0000040
Traes_6BL_F4597CA77.1	secologanin synthase	Infinite	0·0000040
Traes_7AL_DE79AE0D6.1	secologanin synthase-like	Infinite	1·12E-05
Traes_1DL_0DF53C55A.1	secologanin synthase	37·70	0·0012
Traes_6BL_5B70744B1.2	secologanin synthase	24·69	0·00016
Traes_7AL_E70933BD3.1	secologanin synthase-like	9·82	2·27E-07
Traes_1AL_E1F3614B3.1	secologanin synthase-like	9·75	1·45E-14
Traes_5DL_A75494E29.1	secologanin synthase	8·59	0·0040
Traes_4DL_B21B78908.1	secologanin synthase-like	8·52	0·011
Traes_1BL_9B3C88F90.1	secologanin synthase	5·23	0·00069
Traes_6DL_C26461F78.1	secologanin synthase	5·05	0·026
Traes_7DL_2EDF5CB11.1	secologanin synthase-like	4·60	8·53E-12
Traes_7BL_EF7F31461.1	secologanin synthase-like	2·69	0·002

## CONCLUSIONS

Overall, the observed host response in bread wheat to *F. pseudograminearum* during early infection exhibited enrichment of processes related to pathogen perception, defence signalling, transport and metabolism, and deployment of chemical and enzymatic defences. To date, the molecular mechanisms by which wheat perceives *F. pseudograminearum* and mounts a response are still poorly understood. In this study, observation of global transcriptional changes during early infection facilitated identification of defence-related metabolites induced in response to Fusarium crown rot. Exploiting defence-related metabolite pathways may produce tractable resistance in cereal crops to necrotrophic fungal pathogens by increasing accumulation levels of metabolites (Jirschitzka *et al.*, 2013). Given that wheat is susceptible to Fusarium crown rot, some of the genes and signalling pathways identified here may also be acting as susceptibility factors. Additional functional analyses are required to determine the exact roles of these genes in disease resistance or susceptibility. Nevertheless, coupling transcriptomic and metabolite analyses to identify genes and pathways will aid the development of future disease improvement strategies against this important plant pathogen.

## SUPPLEMENTARY DATA

Supplementary data are available online at www.aob.oxfordjournals.org and consist of the following. File S1: relative expression of *F. pseudograminearum* responsive genes across an infection time-course using qRT-PCR. File S2: primers used for qRT-PCR validation of differential gene expression. File S3: read counts generated from individual sequencing libraries. File S4: development of *Tri5* knockout mutants in *F. pseudograminearum*. File S5: multiple reaction monitoring transitions used for quantification of MBOA, tryptamine, serotonin and secologanin using LC-MS. File S6: genes differentially expressed (>2-fold) between mock- and *F. pseudograminearum*-inoculated samples determined by RNA-seq analysis. File S7: gene ontology terms enriched within the set of differentially expressed genes. File S8: phylogenetic tree displaying the relationship between up-regulated wheat UDP-glycosyltransferase genes in wheat and known DON detoxifying genes in *Brachypodium* and barley. File S9: quantification of 6-methoxy-2-benzoxazalinone in wheat seedlings across an *F. pseudograminearum* infection time-course using LC-MS. File S10: quantification of secologanin in wheat seedlings across an *F. pseudograminerum* infection time-course using LC-MS. Table S1: differentially expressed pathogenesis-related genes. Table S2: differentially expressed oxalate oxidase and germin-like protein encoding genes. Table S3: differentially expressed disease resistance protein encoding genes. Table S4: differentially expressed transcription factor genes. Table S5: differentially expressed ABC transporter encoding genes. Table S6: differentially expressed aminocyclopropane carboxylate oxidase genes. Table S7: differentially expressed cytokinin-*o*-glucosyltransferase genes. Table S8: differentially expressed cytochrome p450 genes. Table S9: differentially expressed terpene synthase encoding genes.

## Supplementary Material

Supplementary Data
