# Supplementary material for: The Fusarium crown rot pathogen Fusarium pseudograminearum triggers a suite of transcriptional and metabolic changes in bread wheat (Triticum aestivum L.)
Source: Ann Bot. 2016 Dec 7;119(5):853–67. doi: 10.1093/aob/mcw207 (PMC5604588; doi:10.1093/aob/mcw207)
Supplement: Supplementary Data [file mcw207_Supp.zip › aob-16179-s11.docx]

Supplementary Table 1. Differentially expressed pathogenesis related genes displaying chromosomal survey sequence gene ID, BLAST2GO inferred gene description, differential expression fold-change and adjusted *p*-value.

| Gene ID | Gene Description (BLAST2GO) | DE Fold Change | *p*-adj |
| --- | --- | --- | --- |
| Traes_5DL_43D95A3FE.1 | pathogenesis-related protein 1 | Inf | 0.0018 |
| Traes_5BL_E0E3EC75D.1 | pathogenesis-related protein 1 | 191.65 | 0.00080 |
| Traes_2DL_D267A495A.1 | pathogenesis-related protein 5 | 102.06 | 0.0037 |
| Traes_7DL_0FD6D8ED61.2 | pathogenesis-related protein 5 | 90.40 | 2.79E-20 |
| Traes_4AL_1F77AED931.1 | pathogenesis-related protein 5 | 89.33 | 0.027 |
| Traes_7BS_AB98E8571.1 | pathogenesis-related protein prms | 66.07 | 9.06E-06 |
| Traes_7DS_10C38526F1.1 | pathogenesis-related protein 1 | 56.05 | 0.019 |
| Traes_7DS_A6D32CAA9.2 | pathogenesis related protein- | 32.02 | 2.85E-11 |
| Traes_5DS_8F312CB951.3 | pathogenesis-related protein 10 | 23.43 | 1.54E-05 |
| Traes_7DS_ABBDA3DBB.1 | pathogenesis-related protein prb1-2-like | 23.20 | 1.05E-05 |
| Traes_7BS_94EB3B3D6.1 | pathogenesis-related protein prms | 20.65 | 1.44E-06 |
| Traes_7DS_5969C1C34.1 | pathogenesis-related protein prms | 17.63 | 0.0010 |
| Traes_6AL_E3AACC03A.1 | pathogenesis-related protein 4 | 14.74 | 0.0097 |
| Traes_7DS_1797D4951.1 | pathogenesis-related protein prb1-2-like | 14.12 | 0.0073 |
| Traes_7AS_406885D49.1 | pathogenesis-related protein prb1-2-like | 13.59 | 0.00068 |
| Traes_7BS_8D9D397291.1 | pathogenesis related protein- | 13.04 | 0.049 |
| Traes_5AS_FAD05211F.1 | pathogenesis-related protein 10 | 12.01 | 0.014 |
| Traes_7AS_FEE6CC75E1.2 | pathogenesis-related protein prms | 10.96 | 0.003 |
| Traes_6DL_B9F81F7EC.1 | pathogenesis-related protein 4 | 9.22 | 0.007 |
| Traes_4AL_586359D761.2 | pathogenesis-related thaumatin-like protein | 8.22 | 0.025 |
| Traes_1BS_2CDDD5B91.1 | pathogenesis-related protein 1 | 7.53 | 0.022 |
| Traes_1DL_D5512C9E1.1 | pathogenesis related protein | 5.36 | 0.00079 |
| Traes_5BS_91BD3E004.1 | pathogenesis-related protein 10 | 5.16 | 0.029 |
| Traes_4AS_C5AE1BBDD.1 | pathogenesis-related protein 1 | 5.07 | 0.00022 |
| Traes_4DS_8D68714BE.1 | pathogenesis-related thaumatin-like protein | 3.35 | 0.036 |
| Traes_5AS_381655EE4.2 | pathogenesis-related protein 5 | 3.05 | 1.69E-05 |
|  |  |  |  |
| Traes_1BL_A5FCA9094.1 | chitinase 8 | Inf | 2.74E-10 |
| Traes_1DL_4EF40E197.1 | chitinase 8 | 147.98 | 1.41E-12 |
| Traes_1AL_E96C0662D.2 | chitinase 8 | 115.20 | 4.15E-12 |
| Traes_1BL_265653FAF.1 | chitinase 8 | 46.84 | 4.24E-10 |
| Traes_1DL_95936DC50.1 | chitinase 8 | 34.81 | 4.75E-10 |
| Traes_2AL_225DFC16A.1 | chitinase 5 | 7.77 | 7.39E-11 |
| Traes_2BL_2D440C559.1 | chitinase 5 | 6.86 | 5.22E-09 |
| Traes_2BL_E366A5226.1 | chitinase 5-like | 2.68 | 7.92E-07 |
| Traes_2DL_1CB540BCC.3 | chitinase 5-like | 2.60 | 0.00035 |

Supplementary Table 2. Differentially expressed oxalate oxidase and germin-like protein encoding genes displaying chromosomal survey sequence gene ID, BLAST2GO inferred gene description, differential expression fold-change and adjusted *p*-value.

| Gene ID | Gene Description (BLAST2GO) | DE Fold Change | *p*-adj |
| --- | --- | --- | --- |
| Traes_4BS_7AE61936D.1 | oxalate oxidase gf- | 9.10 | 1.73E-09 |
| Traes_4DS_63083B4941.1 | oxalate oxidase gf- | 9.09 | 4.47E-20 |
| Traes_1DS_123194D79.1 | oxalate oxidase gf- | 8.79 | 5.69E-05 |
| Traes_4AL_B61DB56E6.1 | oxalate oxidase gf- | 8.64 | 1.26E-31 |
| Traes_4AL_E96501F35.1 | oxalate oxidase gf- | 5.52 | 1.58E-06 |
| Traes_4AL_71CD6D929.1 | oxalate oxidase gf- | 4.86 | 0.00010 |
| Traes_4BS_DA73ED408.2 | oxalate oxidase gf- | 4.86 | 0.00000016 |
| Traes_4BS_DA73ED408.2 | oxalate oxidase gf- | 4.86 | 0.00000016 |
| Traes_4DS_ABCFAB135.1 | oxalate oxidase 2 | 4.66 | 0.011 |
| Traes_4DS_8721A5B16.1 | oxalate oxidase gf- | 4.41 | 6.86E-12 |
| Traes_4DS_B5A1413E1.1 | oxalate oxidase gf- | 4.18 | 9.52E-28 |
| Traes_4AL_5CC914E5A.1 | oxalate oxidase gf- | 4.05 | 0.00000014 |
| Traes_4AL_5CC914E5A.1 | oxalate oxidase gf- | 4.05 | 0.00000014 |
| Traes_4BS_40CFAA1A8.1 | oxalate oxidase gf- | 4.02 | 7.30E-05 |
| Traes_4DS_92795CFC91.1 | oxalate oxidase gf- | 3.86 | 9.38E-08 |
| Traes_4DS_90830E0B2.1 | oxalate oxidase gf- | 3.63 | 2.98E-11 |
| Traes_4DS_1684EB8521.1 | oxalate oxidase gf- | 3.13 | 3.23E-09 |
| Traes_4BS_9BDCDFB99.1 | oxalate oxidase gf- | 2.77 | 2.24E-05 |
| Traes_4DS_805590E76.1 | oxalate oxidase gf- | 2.62 | 3.04E-08 |
| Traes_3B_1962330BB.1 | oxalate oxidase gf- | 2.20 | 9.90E-05 |
| Traes_3B_13E045F8D.1 | oxalate oxidase gf- | 2.03 | 0.000000096 |
| Traes_3B_13E045F8D.1 | oxalate oxidase gf- | 2.03 | 0.000000096 |
|  |  |  |  |
| Traes_4BS_7D68271D6.1 | germin-like protein 8-5 | Inf | 0.025 |
| Traes_4BS_48B386606.1 | germin-like protein 8-5 | Inf | 0.032 |
| Traes_5BL_A11EFC122.1 | germin-like protein 8-5 | Inf | 2.27E-10 |
| Traes_4DL_8E52A4172.2 | germin-like protein 8-5 | 34.35 | 5.86E-13 |
| Traes_5BL_A1E29FE1C.1 | germin-like protein 8-5 | 33.99 | 0.0033 |
| Traes_4BL_03873D07C.2 | germin-like protein 8-5 | 28.75 | 0.00014 |
| Traes_2AS_298F369BE.2 | germin-like protein 8-5 | 25.47 | 4.20E-07 |
| Traes_2AS_056ADB1BE.1 | germin-like protein 8-5 | 18.50 | 6.93E-06 |
| Traes_6AL_2BA9DA334.1 | germin-like protein 8-5 | 15.88 | 2.46E-07 |
| Traes_5AL_A3CF2AB1B.1 | germin-like protein 8-5 | 14.52 | 0.0086 |
| Traes_5BL_67F01B7FA.1 | germin-like protein 8-5 | 11.35 | 0.00013 |
| Traes_5BL_72B476ADC.1 | germin-like protein 8-5 | 7.61 | 2.08E-09 |
| Traes_4BL_30DF28795.1 | germin-like protein 8-5 | 7.23 | 0.00052 |
| Traes_6DL_A7FFC6CC3.1 | germin-like protein 1-1 | 6.52 | 0.023 |
| Traes_5BL_B92355534.1 | germin-like protein subfamily t member 2-like | 4.27 | 1.27E-08 |
| Traes_4BL_2A8052BFD.1 | germin-like protein 8-5 | 3.96 | 0.0038 |
| Traes_6DS_27A873663.1 | germin-like protein subfamily 3 member 2 | 2.61 | 0.00018 |
| Traes_6DS_55790D2FA.1 | germin-like protein subfamily 3 member 2-like | 2.06 | 3.37E-11 |

Supplementary Table 3. Differentially expressed disease resistance protein encoding genes displaying chromosomal survey sequence gene ID, BLAST2GO inferred gene description, differential expression fold-change and adjusted *p*-value.

| Gene ID | Gene Description (BLAST2GO) | DE Fold Change | *p*-adj |
| --- | --- | --- | --- |
| Traes_5DL_828B0D1D0.1 | disease resistance response protein 206-like | Inf | 0.022 |
| Traes_5BL_0A5F46BD9.1 | disease resistance response protein 206-like | 12.75 | 0.00041 |
| Traes_1DS_FB5D75ECD.1 | disease resistance protein rpm1 | 8.18 | 0.0058 |
| Traes_6BS_B0E9CDCB7.2 | disease resistance protein rpm1 | 6.88 | 2.13E-13 |
| Traes_6AS_45D1CB908.1 | disease resistance protein rpm1 | 5.94 | 7.80E-06 |
| Traes_2AL_7C00D5F70.2 | disease resistance protein rga1-like | 4.75 | 0.036 |
| Traes_5AL_327F16598.2 | disease resistance protein rpm1 | 3.92 | 0.035 |
| Traes_1AS_AAB89883E1.1 | disease resistance protein rpm1 | 3.40 | 0.0046 |
| Traes_2DL_37D967CFC.2 | disease resistance protein rps2 | 3.05 | 0.000035 |
| Traes_2DL_37D967CFC.2 | disease resistance protein rps2 | 3.05 | 0.000035 |
| Traes_5BL_92A6965F2.1 | disease resistance protein rga3-like | 2.49 | 0.047 |
| Traes_6BS_DBD494D6D.2 | disease resistance protein rga2 | 2.04 | 0.038 |
|  |  |  |  |
| Traes_2AS_E92C7ABDF.1 | mlo-like protein 1 | 20.47 | 0.0036 |
| Traes_2BS_3F2EDD7AB.2 | mlo-like protein 1 | 16.79 | 8.73E-07 |
| Traes_2DS_01D75F7C6.1 | mlo-like protein 1 | 13.99 | 0.000033 |
| Traes_2DS_01D75F7C6.1 | mlo-like protein 1 | 13.99 | 0.000033 |
| Traes_2BS_DABEABDDC.1 | mlo-like protein 1 | 13.59 | 0.0035 |
| Traes_2DS_DC6CAE528.1 | mlo-like protein 1 | 7.20 | 0.0065 |
| Traes_2AS_1FC67C1BD.1 | mlo-like protein 1-like | 3.84 | 2.89E-13 |
| Traes_4DL_1FD62D6E2.1 | mlo protein | 3.76 | 0.022 |
|  |  |  |  |
| Traes_3DS_024168739.1 | rust resistance kinase lr10 | 4.24 | 6.13E-05 |
| Traes_3B_8F4851493.2 | rust resistance kinase lr10 | 3.93 | 1.46E-05 |
| Traes_3AL_5CC7A7B68.4 | wheat leaf rust resistance lr21 | 2.13 | 0.00020 |

Supp. Table 4. Differentially expressed transcription factor genes displaying chromosomal survey sequence gene ID, BLAST2GO inferred gene description, differential expression fold-change and adjusted *p*-value.

| Gene ID | Gene Description (BLAST2GO) | DE Fold Change | *p*-adj |
| --- | --- | --- | --- |
| Traes_2BS_3212EB7DF.1 | transcription initiation factor tfiid subunit 3-like | Inf | 0.044 |
| Traes_5DL_0924913F8.1 | transcription factor jungbrunnen 1-like | 35.38 | 0.017 |
| Traes_5BL_3900B9128.1 | transcription factor jungbrunnen 1-like | 26.28 | 0.0036 |
| Traes_3B_41047D5E6.2 | wrky transcription factor partial | 23.34 | 0.0037 |
| Traes_2DL_362A1F535.1 | wrky45-like transcription factor | 11.41 | 3.27E-06 |
| Traes_3B_7F3B35623.1 | wrky transcription factor | 9.96 | 0.0019 |
| Traes_1AL_9ADA7A031.1 | wrky transcription factor partial | 9.87 | 4.13E-09 |
| Traes_5BL_8BEF7F9CD.1 | wrky transcription factor 40 | 9.77 | 4.14E-05 |
| Traes_1DL_D550418641.2 | wrky transcription factor partial | 8.35 | 0.042 |
| Traes_2DL_B8483F711.1 | wrky45-like transcription factor | 6.64 | 0.0028 |
| Traes_7DL_A9EF00572.1 | wrky transcription factor 70 | 6.23 | 0.0060 |
| Traes_7AS_C9DF68E53.2 | wrky transcription factor 72 | 5.41 | 0.014 |
| Traes_1AL_7BE5906B2.1 | ethylene-responsive transcription factor 5-like | 5.33 | 0.0042 |
| Traes_7DL_A8C4FFABB.1 | nac domain transcription factor superfamily protein | 4.51 | 0.034 |
| Traes_6DS_8F684013D.2 | wrky transcription factor | 4.14 | 7.03E-05 |
| Traes_5BL_4497A137C.1 | nac transcription factor 2b | 3.02 | 5.87E-10 |
| Traes_7DL_B09854286.1 | wrky transcription factor | 2.97 | 9.97E-05 |
| Traes_3DL_2795DC25B.1 | transcription factor bhlh041-like | 2.85 | 0.047 |
| Traes_5DL_C7DA1DA6F.1 | transcription factor jungbrunnen 1-like | 2.85 | 3.13E-10 |
| Traes_6AS_DA75BB1FD.1 | wrky transcription factor | 2.62 | 3.38E-07 |
| Traes_3B_990298FF5.1 | wrky transcription factor 33 | 2.57 | 1.16E-10 |
| Traes_3AL_516137588.1 | transcription factor bhlh041-like | 2.51 | 1.41E-07 |
| Traes_3DL_5F3D20D9F.1 | transcription factor bhlh128-like | 2.48 | 0.047 |
| Traes_6DS_7DD7EDF01.1 | wrky transcription factor | 2.47 | 0.019 |
| Traes_5BL_39FF03C5D.1 | transcription factor jungbrunnen 1-like | 2.44 | 0.0025 |
| Traes_3AL_0C7CB044E.1 | wrky transcription factor 33 | 2.42 | 0.0082 |
| Traes_5BL_F80E01D65.1 | heat stress transcription factor b-1-like | 2.25 | 0.017 |
| Traes_5DL_431CCA490.1 | heat stress transcription factor b-1-like | 2.14 | 8.11E-07 |
| Traes_7AL_C3FEBECBC.1 | wrky transcription factor 40 | 2.08 | 0.042 |
| Traes_5BL_65826E1A1.1 | heat stress transcription factor b-1-like | 2.02 | 0.030 |

Supplementary Table 5. Differentially expressed ABC transporter encoding genes displaying chromosomal survey sequence gene ID, BLAST2GO inferred gene description, differential expression fold-change and adjusted *p*-value.

| **Gene ID** | **Gene Description (BLAST2GO)** | **DE Fold Change** | ***p*-adj** |
| --- | --- | --- | --- |
| **Traes_5BL_4C97C5DE4.1** | pleiotropic drug resistance protein 5-like | Inf | 0.0025 |
| **Traes_3DL_365F92C64.1** | pdr-like abc transporter | 34.90 | 2.82E-18 |
| **Traes_3AL_EEA2DF5E4.1** | pleiotropic drug resistance protein 4-like | 33.24 | 0.00016 |
| **Traes_4AL_70CC7A646.1** | pleiotropic drug resistance protein 5-like | 14.69 | 1.67E-10 |
| **Traes_3B_E1D9AD646.1** | pleiotropic drug resistance protein 4 | 10.49 | 1.12E-09 |
| **Traes_7DL_CCE27DF79.2** | pleiotropic drug resistance protein 4 | 10.10 | 1.43E-13 |
| **Traes_5BL_AAE5B3832.1** | pleiotropic drug resistance protein 5-like | 8.03 | 0.00039 |
| **Traes_5AL_F8FDAC215.2** | pleiotropic drug resistance protein 5 | 5.57 | 0.000000001 |
| **Traes_5AL_F8FDAC215.2** | pleiotropic drug resistance protein 5 | 5.57 | 0.000000001 |
| **Traes_7AL_7E1C0EDEE.1** | pleiotropic drug resistance protein 4 | 5.42 | 5.31E-05 |
| **Traes_7BL_D3A25B6C7.2** | pleiotropic drug resistance protein 4 | 5.25 | 9.02E-06 |
| **Traes_5DL_A7D264077.1** | pleiotropic drug resistance protein 5 | 4.81 | 2.31E-07 |
| **Traes_5DL_F292F9EA4.1** | pleiotropic drug resistance protein 5 | 4.62 | 1.15E-07 |
| **Traes_3DS_294EA7477.1** | abc transporter c family member 3 | 4.48 | 0.0075 |
| **Traes_7AL_A29227860.2** | pleiotropic drug resistance protein 4 | 2.89 | 0.0018 |
| **Traes_3AL_8A13B22C7.1** | pleiotropic drug resistance protein 4 | 2.83 | 1.02E-05 |
| **Traes_3DL_920C6D20E.2** | pdr-like abc transporter | 2.78 | 1.69E-10 |
| **Traes_3B_4434107C4.1** | pleiotropic drug resistance protein 4 | 2.78 | 4.36E-13 |
| **Traes_3AL_0C1B383C3.1** | pleiotropic drug resistance protein 4-like | 2.74 | 4.86E-10 |
| **Traes_5AS_D714D4951.1** | pleiotropic drug resistance protein 5 | 2.73 | 1.89E-05 |
| **Traes_3DS_EE5D85DE2.1** | abc transporter c family member 3-like | 2.61 | 6.31E-10 |
| **Traes_1DL_A6FE85E09.1** | abc transporter a family member 7-like | 2.59 | 0.041 |
| **Traes_1AL_AAB83FA9B.1** | abc transporter a family member 2-like | 2.41 | 0.00035 |
| **Traes_2AL_FAA384931.2** | pleiotropic drug resistance protein 4 | 2.39 | 0.044 |
| **Traes_3B_984F0A188.2** | pleiotropic drug resistance protein 4 | 2.37 | 0.0015 |
| **Traes_7DL_AEAE90568.1** | pdr-like abc transporter | 2.33 | 2.24E-05 |
| **Traes_7DS_D89F89766.1** | pleiotropic drug resistance protein 2-like | 2.30 | 0.0058 |
| **Traes_7DS_EB0ADD31F.1** | abc transporter c family member 10 | 2.27 | 3.67E-05 |
| **Traes_4AL_603B6DC64.1** | pleiotropic drug resistance protein 3-like | 2.16 | 0.0019 |
| **Traes_7DL_A95F658D8.2** | abc transporter c family member 8-like | 2.11 | 0.0074 |
| **Traes_7AS_14DE5D999.2** | abc transporter c family member 10 | 2.01 | 0.00024 |
| **Traes_7BS_CF247665F.1** | pleiotropic drug resistance protein 2-like | 2.00 | 0.0068 |
| **Traes_6DL_1336419B9.1** | abc transporter b family member 2-like | 0.48 | 2.44E-06 |
| **Traes_3AS_98C9AD113.1** | abc transporter g family member 32 | 0.45 | 0.012 |

Supplementary Table 6. Differentially expressed aminocyclopropane carboxylate oxidase genes displaying chromosomal survey sequence gene ID, BLAST2GO inferred gene description, differential expression fold-change and adjusted *p*-value.

| **Gene ID** | **Gene Description (BLAST2GO)** | **DE Fold Change** | ***p*-adj** |
| --- | --- | --- | --- |
| **Traes_2AS_48A3F9466.1** | 1-aminocyclopropane-1-carboxylate oxidase-1-like protein | 9.27 | 0.0073 |
| **Traes_1AS_ED61D0E0C.1** | acc oxidase | 3.18 | 1.29E-08 |
| **Traes_1AS_C2D84E4F8.1** | acc oxidase | 2.88 | 0.00014 |
| **Traes_1AS_A2C4350B8.1** | acc oxidase | 2.77 | 0.00075 |
| **Traes_2BS_CF4F892DD.1** | 1-aminocyclopropane-1-carboxylate oxidase-1-like protein | 2.56 | 0.043 |
| **Traes_1BS_C428DA20B.1** | acc oxidase | 2.51 | 0.00065 |
| **Traes_1DS_AEB0CD565.1** | acc oxidase | 2.44 | 0.0038 |
| **Traes_1AS_A12B2DC63.1** | acc oxidase | 2.39 | 0.0013 |
| **Traes_1DS_3463BE1BB.1** | acc oxidase | 2.34 | 0.0060 |
| **Traes_1BS_4F863C0A0.1** | acc oxidase | 2.28 | 2.53E-08 |
| **Traes_1DS_99E3E8761.1** | acc oxidase | 2.26 | 0.00012 |
| **Traes_1DS_26E6C748F.1** | acc oxidase | 2.02 | 0.00061 |

Supplementary Table 7. Differentially expressed cytokinin-o-glucosyltransferase genes displaying chromosomal survey sequence gene ID, BLAST2GO inferred gene description, differential expression fold-change and adjusted *p*-value.

| Gene ID | Gene Description (BLAST2GO) | DE Fold Change | *p*-adj |
| --- | --- | --- | --- |
| Traes_3B_4887B0D241.1 | cytokinin-o-glucosyltransferase 1 | Inf | 0.019 |
| Traes_3B_E9CFE37D3.1 | cytokinin-o-glucosyltransferase 1 | 18.57 | 0.0049 |
| Traes_6DL_8FCA98FC5.1 | cytokinin-o-glucosyltransferase 2 | 18.17 | 0.0050 |
| Traes_6AL_CD93AD097.1 | cytokinin-o-glucosyltransferase 2 | 17.66 | 0.0051 |
| Traes_3B_B021B5C15.1 | cytokinin-o-glucosyltransferase 1 | 13.57 | 0.00052 |
| Traes_2DS_DBE32D98B.1 | cytokinin-o-glucosyltransferase 3 | 12.63 | 2.63E-09 |
| Traes_2AS_5EBF8EF1E.1 | cytokinin-o-glucosyltransferase 2 | 9.56 | 2.22E-25 |
| Traes_6DS_489CD9911.1 | cytokinin-o-glucosyltransferase 2 | 6.63 | 9.90E-05 |
| Traes_3DS_BD8671794.1 | cytokinin-o-glucosyltransferase 1 | 5.13 | 0.011 |
| Traes_3B_E5B805A13.2 | cytokinin-o-glucosyltransferase 1 | 4.73 | 1.09E-07 |
| Traes_3DS_9897882C9.1 | cytokinin-o-glucosyltransferase 1 | 4.07 | 5.83E-05 |
| Traes_6DS_EC682031E.1 | cytokinin-o-glucosyltransferase 2 | 4.03 | 2.16E-15 |
| Traes_3DS_1A4FE3449.1 | cytokinin-o-glucosyltransferase 1 | 3.91 | 0.0053 |
| Traes_6AL_AE7EF8E87.2 | cytokinin-o-glucosyltransferase 2 | 3.34 | 2.83E-06 |
| Traes_3AS_4B9462BD0.2 | cytokinin-o-glucosyltransferase 1 | 3.30 | 0.00011 |
| Traes_6AS_8DDE1D849.1 | cytokinin-o-glucosyltransferase 2 | 3.13 | 0.041 |
| Traes_3DS_9E292BCAF.3 | cytokinin-o-glucosyltransferase 1 | 2.96 | 6.39E-11 |
| Traes_3AS_5B258E7EB.2 | cytokinin-o-glucosyltransferase 1 | 2.84 | 0.00026 |

Supplementary Table 8. Differentially expressed cytochrome p450 genes displaying chromosomal survey sequence gene ID, BLAST2GO inferred gene description, differential expression fold-change and adjusted P value.

| Gene ID | Gene Description (BLAST2GO) | DE Fold Change | *p*-adj |
| --- | --- | --- | --- |
| Traes_2DS_0DCF00A43.1 | cytochrome p450 99a2 | Inf | 0.0013 |
| Traes_2DS_936E3050F.1 | cytochrome p450 99a2 | Inf | 0.0024 |
| Traes_2DS_C35431082.1 | cytochrome p450 99a2 | Inf | 0.0036 |
| Traes_2AS_8F32006DE.1 | cytochrome p450 99a2 | Inf | 0.011 |
| Traes_2AS_2B63DDD75.1 | cytochrome p450 99a2 | 19.87 | 0.012 |
| Traes_2BL_27FB0A099.1 | cytochrome p450 99a2 | 16.84 | 0.0025 |
| Traes_2AS_8615CCEF0.1 | cytochrome p450 99a2 | 16.40 | 0.021 |
| Traes_2AS_10D194CC7.1 | cytochrome p450 99a2 | 15.24 | 1.35E-10 |
| Traes_2DS_E47CE2C01.1 | cytochrome p450 99a2 | 10.76 | 0.021 |
| Traes_2AS_51739A52D.1 | cytochrome p450 99a2 | 8.83 | 1.20E-07 |
| Traes_2DS_17780309E.1 | cytochrome p450 71d7 | Inf | 0.011 |
| Traes_2BS_011B0377F.2 | cytochrome p450 71d7 | Inf | 4.93E-05 |
| Traes_2AL_B1E9FF655.1 | cytochrome p450 71d7 | 64.26 | 2.37E-06 |
| Traes_2AS_195870BB8.1 | cytochrome p450 71d7 | 57.62 | 5.58E-06 |
| Traes_2DL_43959DBBD.1 | cytochrome p450 71d7 | 28.14 | 1.88E-05 |
| Traes_5DL_DE930D8C9.2 | cytochrome p450 71d7 | 12.58 | 0.0032 |
| Traes_2BL_85629BCE9.1 | cytochrome p450 71d7 | 11.89 | 8.22E-31 |
| Traes_2DS_3EC0F26D4.1 | cytochrome p450 71d7 | 6.45 | 0.00010 |
| Traes_2DS_9038228DC.1 | cytochrome p450 71d7 | 5.40 | 4.38E-09 |
| Traes_2DS_1E5EB2757.1 | cytochrome p450 71d7 | 5.03 | 1.73E-05 |
| Traes_7AS_93B202809.1 | cytochrome p450 71d10 | Inf | 6.54E-05 |
| Traes_2AL_E0AB32412.1 | cytochrome p450 71d11 | 31.56 | 8.82E-10 |
| Traes_2DL_63CCFFFE4.1 | cytochrome p450 71d11 | 31.41 | 0.034 |
| Traes_5BL_F5F24C495.1 | cytochrome p450 71d11 | 9.81 | 0.019 |
| Traes_5DL_C65153D64.1 | cytochrome p450 71c4 | 56.08 | 2.97E-05 |
| Traes_5BL_3F9D3DFE8.1 | cytochrome p450 71c4 | 12.21 | 8.22E-17 |
| Traes_2AL_53BE4ECB9.1 | cytochrome p450 76c2-like | 54.33 | 1.29E-14 |
| Traes_7BL_5EB114AB7.1 | cytochrome p450 87a3-like | 6.60 | 9.51E-12 |
| Traes_4DL_66BE3C639.2 | cytochrome p450 87a3-like | 5.40 | 1.42E-53 |
| Traes_7DS_7DA7A6420.1 | cytochrome p450 | 4.79 | 0.00032 |
| Traes_5BL_6240DE6BA.2 | cytochrome p450 | 2.96 | 3.49E-08 |
| Traes_5DL_5FCF0AFAC.1 | cytochrome p450 | 2.96 | 0.0049 |
| Traes_5BL_4AB318AA4.1 | cytochrome p450 | 2.09 | 0.020 |
| Traes_3AS_D6DD0F6B9.2 | cytochrome p450 734a6-like | 0.42 | 0.0099 |
| Traes_7DS_2BAF96919.1 | cytochrome p450 superfamily protein | 0.41 | 0.018 |
| Traes_1DL_E2F8387A4.1 | cytochrome p450 71a1 | 0.34 | 0.046 |

Supplementary Table 9. Differentially expressed terpene synthase encoding genes displaying chromosomal survey sequence gene ID, BLAST2GO inferred gene description, differential expression fold-change and adjusted *p*-value.

| **Gene ID** | **Gene Description (BLAST2GO)** | **DE Fold Change** | ***p*-adj** |
| --- | --- | --- | --- |
| **Traes_2AS_1569FE9F11.1** | syn-copalyl diphosphate synthase | Inf | 4.19E-07 |
| **Traes_2DS_7917F0E44.1** | syn-copalyl diphosphate synthase | 46.9629622 | 0.031 |
| **Traes_2AS_1569FE9F1.1** | syn-copalyl diphosphate synthase | 33.1900954 | 0.0022 |
| **Traes_2BL_80AC86A1A.1** | syn-copalyl diphosphate synthase | 23.6033471 | 0.0027 |
| **Traes_2DS_22A94EEA6.1** | syn-pimara- -diene synthase | 32.968317 | 9.98E-06 |
| **Traes_2AS_5F7EC1988.1** | syn-pimara- -diene synthase | 20.5673267 | 6.31E-10 |
| **Traes_2AS_2610FD515.1** | syn-pimara- -diene synthase | 16.9970473 | 0.036 |
| **Traes_2AS_D3212BACF.1** | syn-pimara- -diene synthase | 11.8537628 | 2.18E-06 |
