# Supplementary figures and images for: The Fusarium crown rot pathogen Fusarium pseudograminearum triggers a suite of transcriptional and metabolic changes in bread wheat (Triticum aestivum L.)
Source: Ann Bot. 2016 Dec 7;119(5):853–67. doi: 10.1093/aob/mcw207 (PMC5604588; doi:10.1093/aob/mcw207)

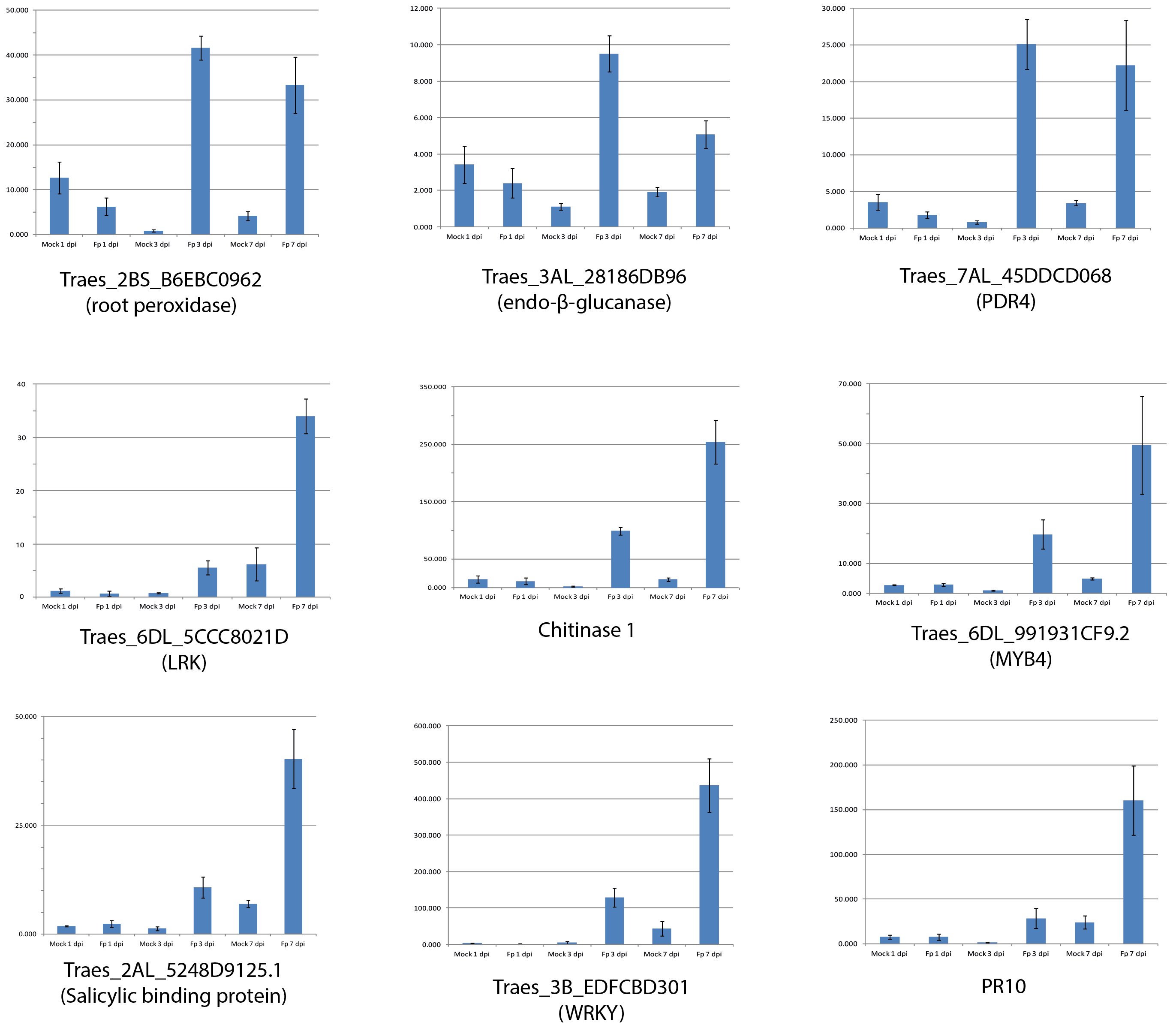

Supplement: Supplementary Data [file mcw207_Supp.zip › aob-16179-s02.jpg]

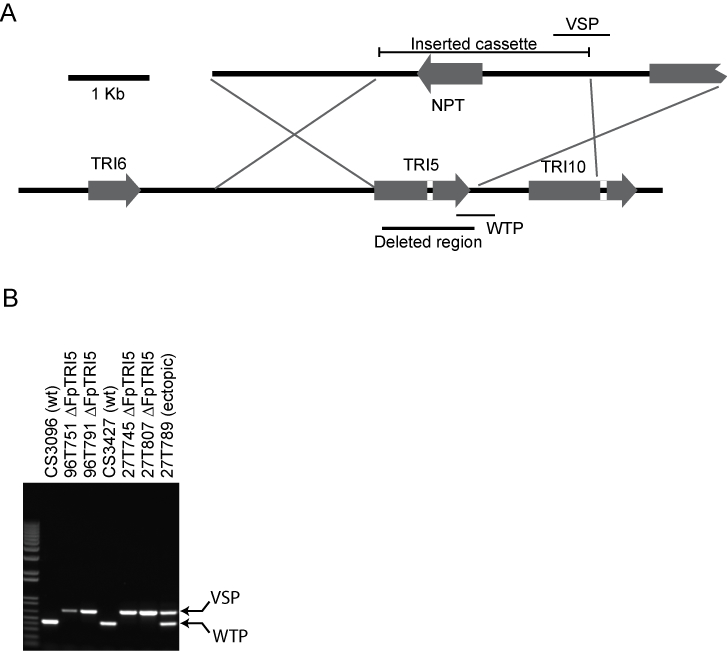

Supplement: Supplementary Data [file mcw207_Supp.zip › aob-16179-s05.jpg]

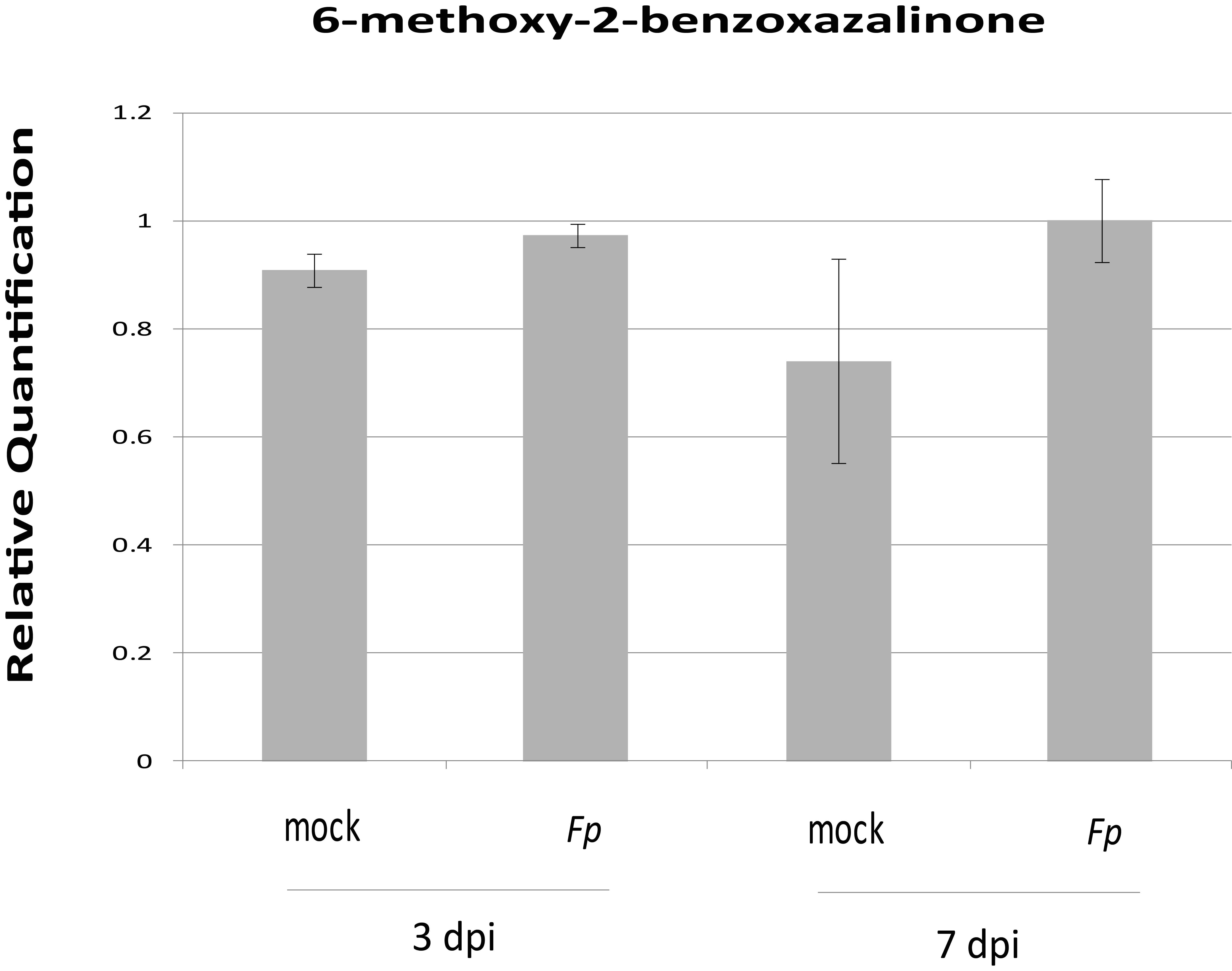

Supplement: Supplementary Data [file mcw207_Supp.zip › aob-16179-s09.jpg]

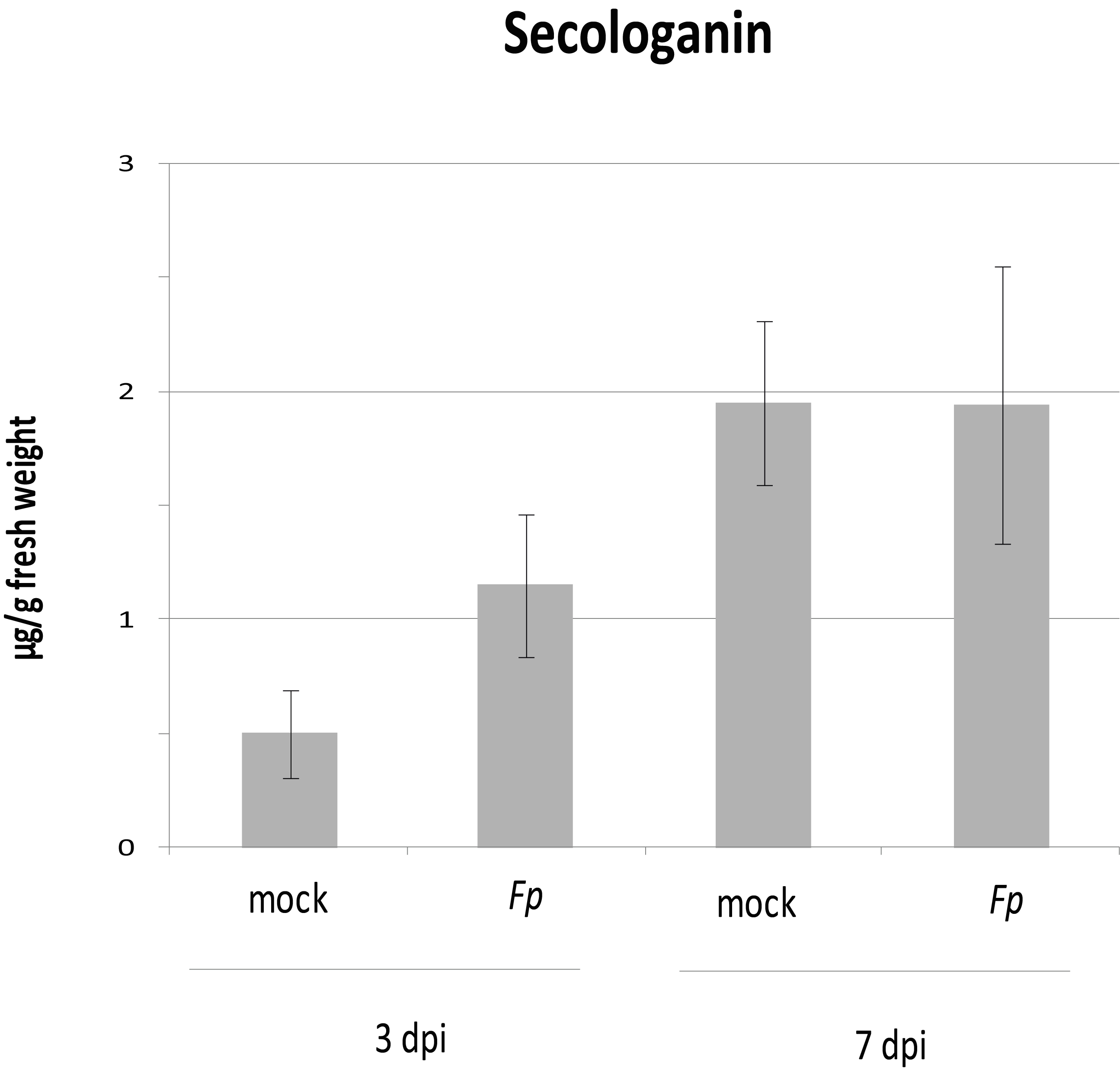

Supplement: Supplementary Data [file mcw207_Supp.zip › aob-16179-s10.jpg]
